# A Glb1-2A-mCherry reporter monitors systemic aging and predicts lifespan in middle-aged mice

**DOI:** 10.1038/s41467-022-34801-9

**Published:** 2022-11-17

**Authors:** Jie Sun, Ming Wang, Yaqi Zhong, Xuan Ma, Shimin Sun, Chenzhong Xu, Linyuan Peng, Guo Li, Liting Zhang, Zuojun Liu, Ding Ai, Baohua Liu

**Affiliations:** 1 Shenzhen Key Laboratory for Systemic Aging and Intervention (SKL-SAI), School of Basic Medical Sciences, Shenzhen University, Shenzhen, 518055 China; 2 Guangdong Key Laboratory of Genome Stability and Human Disease Prevention, Marshall Laboratory of Biomedical Engineering, National Engineering Research Center for Biotechnology (Shenzhen), International Cancer Center, Shenzhen University, Shenzhen, 518055 China; 3 Shenzhen Bay Laboratory, Shenzhen, China; 4 Department of Dermatology, Xiangya Hospital, Central South University, Changsha, China; 5 Department of Physiology and Pathophysiology, Tianjin Medical University, Tianjin, 300070 China

**Keywords:** Senescence, Single-cell imaging, Ageing

## Abstract

The progressive decline of physiological function and the increased risk of age-related diseases challenge healthy aging. Multiple anti-aging manipulations, such as senolytics, have proven beneficial for health; however, the biomarkers that label in vivo senescence at systemic levels are lacking, thus hindering anti-aging applications. In this study, we generate a *Glb1*^+/m^‒Glb1-2A-mCherry (GAC) reporter allele at the *Glb1* gene locus, which encodes lysosomal β-galactosidase—an enzyme elevated in tissues of old mice. A linear correlation between GAC signal and chronological age is established in a cohort of middle-aged (9 to 13 months) *Glb1*^+/m^ mice. The high GAC signal is closely associated with cardiac hypertrophy and a shortened lifespan. Moreover, the GAC signal is exponentially increased in pathological senescence induced by bleomycin in the lung. Senolytic dasatinib and quercetin (D + Q) reduce GAC signal in bleomycin treated mice. Thus, the Glb1-2A-mCherry reporter mice monitors systemic aging and function decline, predicts lifespan, and may facilitate the understanding of aging mechanisms and help in the development of anti-aging interventions.

## Abstract (web summary)

Monitoring the aging process in vivo is challenging. Here the authors generate a *Glb1*^+/m^‒Glb1-2A-mCherry (GAC) reporter mouse model, where the GAC signal is consistently correlated with established biomarkers of cellular senescence, cardiac hypertrophy and shortened lifespan, which may prove helpful for studies developing anti-aging interventions.

## Introduction

Aging is the progressive deterioration of physiological functions in virtually all tissues and organs and is, thus, the most important risk factor for many diseases, including cardiovascular diseases (CVD), Alzheimer’s disease (AD), osteoporosis, and chronic lung diseases^1–3^. CVD is the leading reason for death in the population of over 65s, as exemplified by cardiopathies and atherosclerosis^4^. Cardiomyocytes are terminally differentiated; in order to adapt to extrinsic and intrinsic stimuli, they increase in size (hypertrophy) rather than number, and sustained cardiac hypertrophy can eventually cause heart failure^5^. Indeed, the hypertrophic growth of cardiomyocytes is a typical characteristic of cardiac aging^6^. In addition, aging is accompanied by a gradual decline in cognition and memory, which are exacerbated in AD and employed as early diagnostic markers^7^. Of particular note, AD patients suffer more severe memory loss than they do cognitive deficits, whereas individuals undergoing normal aging exhibit the opposite^8^. Senolytic dasatinib and quercetin eliminate senescent oligodendrocyte progenitor cells and ameliorate inflammation and cognitive deficits in mouse models mimicking AD^9^. Therefore, understanding aging mechanisms and monitoring aging processes may provide the basis for the early diagnosis, prevention, and treatment of aging-related pathologies.

Accumulation of cellular senescence is one of the hallmarks of aging^10^. In the early 1960’s, Hayflick and Moorhead first reported that in vitro cultured human diploid cells have limited doubling capacity, referred to as replicative senescence or the Hayflick limit, which mainly attributes to telomere attrition^11,12^. Oxidative stress, genomic instability, and oncogene activation also induce senescence, called stress-induced senescence (SIS)^13^. Senescent cells tend to gain lysosomal mass and enhance the expression of β-galactosidase protein, the activity of which can be detected at a suboptimal condition, i.e., at pH 6.0^14^, known as senescence-associated β-galactosidase activity at pH 6.0 (SAβ-gal). The DNA-damaging reagent bleomycin (BLM) can induce lung epithelial cell senescence marked by SAβ-gal^15^. Senescent cells are also marked by accumulated DNA-damage foci γ-H2AX; the increased expression of senescence-associated secretory phenotype (SASP); and the upregulation of *p16*^*Ink4a*^, *p19*^*Arf*^, and *p21*^*Wif1*^ gene transcription^16,17^. A landmark work by Baker et al. demonstrated that the elimination of high-*p16*^*Ink4a*^ cells with an *INK-ATTAC* cassette ameliorated progeroid features in *BubR1* mutant mice^18^. The clearance of high-*p16*^*Ink4a*^ cells extends the lifespan of naturally aged mice^19^. Grosse recently showed that p16-high cells are mainly liver sinusoid endothelial cells, and elimination of these cells caused fibrosis and health deterioration^20^. Of note, the activity of SAβ-gal was used to activate the senolytic SSK1 to eliminate senescent cells and improve the health of mice^21^.

While the biomarkers of the senescence of in vitro cultured cells has been extensively investigated, finding in vivo biomarker(s) is still challenging. This hinders the application of senolytic-exemplified anti-aging manipulations. Sharpless’s group generated an aging reporter by replacing the *Ink4a* allele with a luciferase cassette to monitor the dynamic expression of *p16*^*Ink4a*^ gene^22^. Intriguingly, high luciferase activity in heterozygous *p16*^+/luc^ mice predicts malignant transformation rather than lifespan. The same group generated a *p16*^tdTom^ model that facilitates the enumeration, isolation, and characterization of individual *p16*^*Ink4a*^ high-expressing cell (tdTom+) and indicates that *p16*^*Ink4a*^-activated cells accumulate with aging and inflammation^23^. Nakanishi’s group generated a p16-CreERT2-tdTomato model, which can help analyze the dynamic properties of *p16*^*Ink4a*^-high cells at single-cell level, and demonstrated that elimination of p16-high cells could ameliorate steatosis and inflammation in the liver of a NASH mouse model^24^. Recently, Xu’s group generated an inducible *p21*-Cre mouse model to monitor and manipulate p21^high^ senescent cells in vivo, and a small population of cells (1.5~10%) were p21^high^ in old mice and intermittent clearance of p21^high^ cells improved physical function. SAβ-gal activity reflects the lysosomal β-D-galactosidase encoded by the *Glb1* gene^25^, so we hypothesized that *Glb1* expression levels could be used as an in vivo indicator of cellular senescence and organ dysfunction. In this study, we thus generate a *Glb1*^+/m^ allele−Glb1-2A-mCherry (GAC) reporter at the *Glb1* locus, with the aim of live-imaging and tracing the aging process at the tissue/organ and systemic levels. Significantly, we reveal that the GAC signal linearly correlates with chronological age when the mice are middle-aged (9–13 months). High GAC signal predicts cardiac hypertrophy and a shortened lifespan during physiological aging. In addition, the GAC signal is exponentially induced by BLM, which cause pathological cellular senescence. Moreover, senolytic dasatinib and quercetin reduces GAC signal in BLM treated mice. Thus, the GAC reporter mice will facilitate the understanding of aging mechanisms and the development of anti-aging interventions that help treat aging-related pathologies.

## Results

### Glb1 level marks cellular senescence

**Fig. 1:**
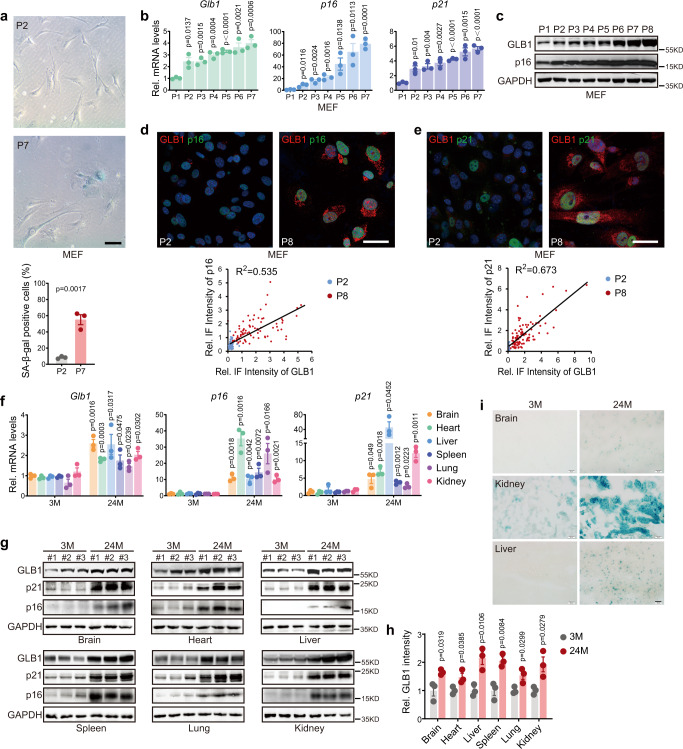
Increased *Glb1* mRNA and protein levels in cell and tissue aging. Increased SAβ-gal with quantification of percent positive cells (bottom panel, over 100 cells per group were counted) and enlarged cell morphology (**a**), elevated mRNA levels of *Glb1*, *p16*^*Ink4a*^, and *p21*^*Wif1*^ (**b**), and upregulated protein levels of GLB1 and p16^Ink4a^ (**c**) in mouse embryonic fibroblasts (MEFs, from *n* = 3 embryos of wild-type C57BL6 mice) during replicative senescence. Scale bar, 100 µm. Representative images showing co-staining of GLB1 (red) and p16^Ink4a^ (green) (**d**, quantification of correlation between two signals in lower panel, over 80 cells per group were counted), and co-staining of GLB1 (red) and p21^Wif1^ (green) (**e**, quantification of correlation between two signals in lower panel, over 100 cells per group were counted) in MEFs during replicative senescence. Scale bar, 50 µm. The mRNA (**f**) and protein (**g**) levels of *Glb1*, *p16*^*Ink4a*^, and *p21*^*Wif1*^ in indicated tissues isolated from young (3 months, male, *n* = 3) and old (24 months, male, *n* = 3) mice. **h** Quantification of GLB1 band intensity in **g**. **i** SAβ-gal staining in brain, kidney, and liver tissues from young (3 months, male, *n* = 3) and old (24 months, male, *n* = 3) mice. Scale bar, 50 µm. Cell passage (P) numbers are indicated. “*n*” represents number of biological replicates. Data represent the means ± s.e.m. *p* value was calculated by Student’s *t* test (two-sided).

Although SAβ-gal has been extensively applied as a cellular senescence marker, its encoding gene, *Glb1*, has been rather less well investigated. Here, we examined the correlation between *Glb1* expression level, β-galactosidase activity, and other cellular senescence markers. In vitro cultured mouse embryonic fibroblasts (MEFs) underwent continuous replicative senescence at passage 7 (P7), as indicated by increased SAβ-gal staining and enlarged cell morphology (Fig. 1a), enhanced *p16*^*Ink4a*^ and/or *p21*^*Wif1*^ mRNA and protein expression (Fig. 1b–e). Consistent with this, the mRNA and protein level of *Glb1* also gradually increased along with passaging. Thus, *Glb1* levels can be used as an alternative cellular senescence marker.

We next studied the level of *Glb1* expression in various tissues obtained from early-age (EA, 3–8 months, represented by 3 months), middle-age (MA, 9–14 months, represented by 12 months), and late-age (LA, 15–27 months, represented by 24 months) mice. Compared with the EA group, *Glb1* mRNA levels in the LA group were significantly upregulated in all tissues examined, including brain, heart, liver, lung, kidney, and spleen (Fig. 1f), which is in line with the upregulation of *p16*^*Ink4a*^ and *p21*^*Wif1*^ in these tissues from LA mice. A similar upregulation of GLB1, p16^Ink4a^ and p21^Wif1^ proteins was observed on western blotting and immunohistochemical (IHC) staining of tissues from LA mice compared with EA mice (Fig. 1g, h and Supplementary Fig. 1). Positive SAβ-gal staining was successfully detected in brain, liver, and kidney tissue sections from the LA group (Fig. 1i), but failed in other tissues. We further examined the levels of *Glb1* in MEFs and human fibroblasts treated with BLM, doxorubicin (DOX), ionizing radiation (IR), hydrogen peroxide (H_2_O_2_), or overexpression of the oncogene Ras, all of which are well-documented senescence inducers. Compared with untreated cells, all the treatment increased mRNA levels of *Glb1*, *p16*^*Ink4a*^ and *p21*^*Wif1*^ in MEFs (Supplementary Fig. 2a). Consistently, determined by western blotting, the protein levels of GLB1, p16^Ink4a^ and p21^Wif1^ were enhanced in MEFs (Supplementary Fig. 2b). Interestingly, in human fibroblast, both H_2_O_2_ and overexpression of Ras upregulated mRNA level of *Glb1*, *p16*^*Ink4a*^ *and p21*^*Wif1*^, but IR only enhanced that of *Glb1*. All the above treatments increased SAβ-gal staining and C12FDG staining determined by FACS (Supplementary Fig. 2c–e). Thus, the level of *Glb1* is largely consistent with current biomarkers of cellular senescence, and can be used as an in vitro and in vivo cellular senescence marker.

### Generation and characterization of Glb1-2A-mCherry (GAC) reporter mice

**Fig. 2:**
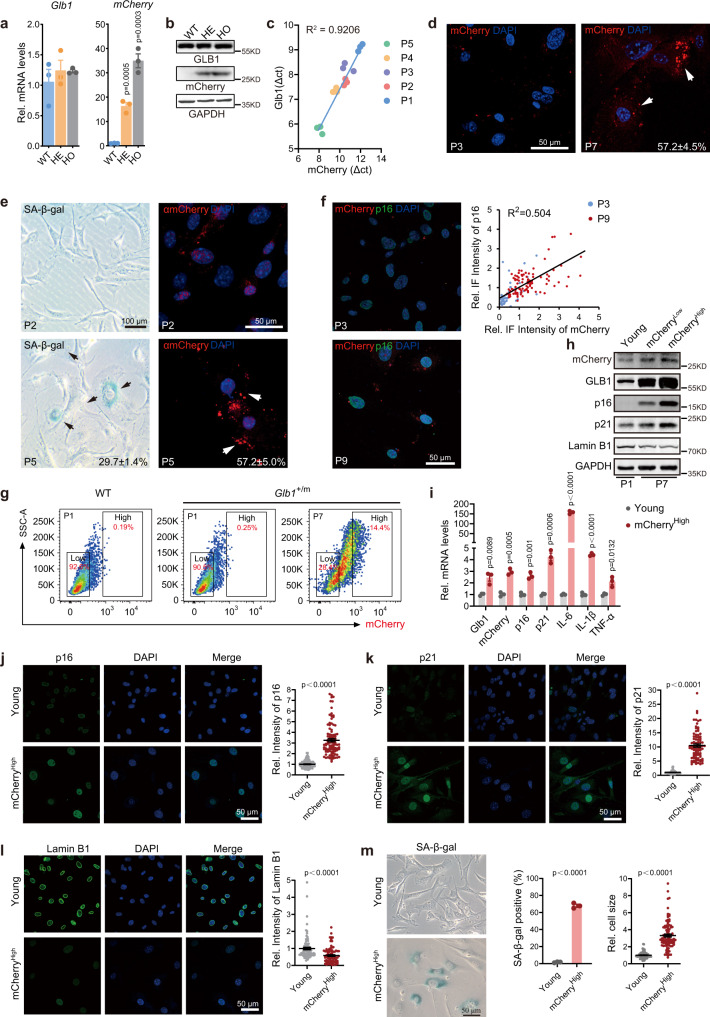
Characterization of GAC reporter at cellular levels. Relative mRNA levels of *Glb1* and *mCherry* (**a**) and representative western blots showing the corresponding protein levels (**b**) in lung tissues from indicated mice. WT, *Glb1*^+/+^ mice (15 months, female, *n* = 3); HE, *Glb1*^+/m^ heterozygous mice (15 months, female, *n* = 3); HO, *Glb1*^m/m^ homozygous mice (15 months, female, *n* = 3). **c** Linear correlation between the mRNA levels of *Glb1* and *mCherry* in passages 1 to 5 of *Glb1*^+/m^ MEF. **d** Representative images showing elevated mCherry fluorescence signal in *Glb1*^+/m^ MEFs at P7. Arrows indicate senescent cells. **e** Representative images showing increased SAβ-gal staining (left) and mCherry protein levels (right) in *Glb1*^+/m^ MEFs at P5. Arrows indicate senescent cells. **f** Representative images showing the co-existence of mCherry signal and anti p16^Ink4a^ fluorescence staining (green) in *Glb1*^+/m^ MEFs during replicative senescence. Relative fluorescence staining intensity was quantified in over 100 cells per group. **g** FACS sorting strategy of mCherry^Low^ and mCherry^High^ *Glb1*^*+/m*^ MEFs. **h** Immunoblotting analysis of mCherry, GLB1 and indicated senescence markers in Young (P1), mCherry^Low^ and mCherry^High^ *Glb1*^*+/m*^ MEFs. **i** qPCR analysis of expression levels of *mCherry*, *Glb1* and indicated senescence marker genes in Young and mCherry^High^ *Glb1*^*+/m*^ MEFs from *n* = 3 embryos. Immunostaining of p16^Ink4a^ (**j**), p21^Wif1^ (**k**) and Lamin B1 (**l**) in Young and mCherry^High^ *Glb1*^*+/m*^ MEFs from *n* = 3 embryos. Relative staining intensity was calculated from over 85 cells per group. **m** SAβ-gal staining in Young and mCherry^High^ *Glb1*^*+/m*^ MEFs from *n* = 3 embryos. Percent SAβ-gal-positive cells and relative cell size was quantified. Over 100 cells per group were counted. Cell passage (P) numbers are indicated. “*n*” represents number of biological replicates. Data represent the means ± s.e.m. *p* value was calculated by Student’s *t* test (two-sided).

To test whether *Glb1* expression levels spatiotemporally indicate physiological aging, we generated a GAC reporter model in C57BL6/J background using the CRISPR/Cas9 system (Supplementary Fig. 3a). Successful knock-in of GAC allele was evidenced by target-specific PCR and sequencing (Supplementary Fig. 3b, c), and the expression of *Glb1* mRNA and protein was not affected (Fig. 2a, b). Both heterozygous and homozygous GAC mice were born at the expected Mendel’s ratio and developed normally into adults (Supplementary Fig. 3d), and their overall lifespan was comparable to that of wild-type littermate mice (see data from later studies).

**Fig. 3:**
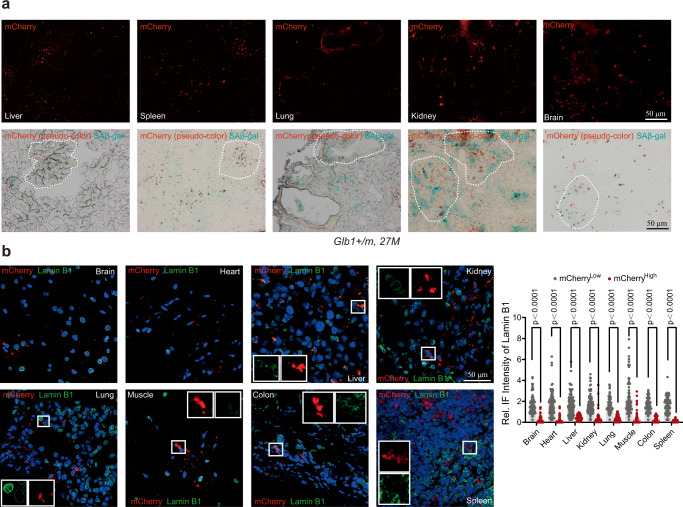
Characterization of GAC reporter at tissue levels. **a** Representative images showing mCherry signal (upper) and its co-localization with SAβ-gal staining (lower, mCherry in pseudo-color) in liver, spleen, lung, kidney and brain tissues from *Glb1*^+/m^ mice (27 months, male, *n* = 3). Dashed polygon indicates area with intense senescence signal. Scale bar, 100 µm. **b** Left, representative images showing mCherry signal and anti-Lamin B1 staining in indicated tissue sections from *Glb1*^+/m^ mice (23 months, female, *n* = 3). The magnified regions show cells with mutually exclusive signals for mCherry and Lamin B1. Right, quantification of mCherry and Lamin B1 signal intensity in indicated tissues (left). Over 100 cells per group were counted. Scale bar, 50 µm. “*n*” represents number of biological replicates. Data represent the means ± s.e.m. *p* value was calculated by Student’s *t* test (two-sided).

The mRNA level of *mCherry* was linearly and significantly correlated with that of *Glb1* in *Glb1*^+/m^ MEFs during continuous culture (Fig. 2c). The protein and/or mRNA levels of *mCherry*, *Glb1*, *p16*^*Ink4a*^, *p21*^*Wif1*^, SASP gene *IL6*, *IL1β* and *TNFα* in late passage (P7) *Glb1*^+/m^ MEFs were significantly upregulated compared to those in early passage (P1) (Supplementary Fig. 4a, b). *Glb1*^+/m^ MEFs that underwent replicative senescence exhibited high level of mCherry fluorescence signal (hereafter referred to as GAC signal) at P7 (Fig. 2d, 57.2 ± 4.5% positive cells), SAβ-gal staining at P5 (Fig. 2e, 29.7 ± 1.4% positive cells), and mCherry protein levels (as detected by immunofluorescence staining with anti-mCherry antibody) at P5 (Fig. 2e, 57.2 ± 5.0% positive cells). Similarly, p16^Ink4a^ was co-stained with mCherry at P9 *Glb1*^+/m^ MEFs (Fig. 2f). To further confirm that the GAC signal labels senescent cells, we sorted the mCherry-high (namely mCherry^High^) and mCherry-low (mCherry^Low^) subpopulations of in vitro cultured *Glb1*^+/m^ MEFs at P1 (young) and P7 by FACS (Fig. 2g). The expression levels of mCherry, GLB1, and the typical senescence marker p16^Ink4a^ and p21^Wif1^ were significantly increased in the mCherry^High^ cells (P7) compared to those in young cells, as determined by western blotting, q-PCR and immunofluorescence staining (Fig. 2h–k and Supplementary Fig. 5a, b). Lamin B1, another senescence marker, was also obviously downregulated in mCherry^High^ cells (Fig. 2h, l). In addition, the mRNA levels of *IL6*, *IL1β* and *TNFα* were significantly upregulated in mCherry^High^ cells (Fig. 2i). The cell size and SAβ-gal staining were also analyzed, and compared to young cells, the size of mCherry^High^ cells was markedly enlarged and most of them were SAβ-gal-positive (Fig. 2m). Further, cell cycle status was determined by anti-Ki67 staining. The majority of mCherry^High^ cells were Ki67-negative/low (Supplementary Fig. 5c), indicating exit from cell cycle. Importantly, GAC signal was detected in multiple tissues of *Glb1*^+/m^ mice, including brain, heart, muscle, liver, lung, spleen, kidney, and colon, especially in cells positive for SAβ-gal (Fig. 3a), or p21^Wif1^ (Supplementary Fig. 6), but negative for Lamin B1 (Fig. 3b). The specificity of the GAC signal to mCherry was confirmed by immunofluorescence and IHC staining with anti-mCherry and/or anti-red fluorescence protein antibodies in all examined tissues of *Glb1*^+/m^ mice (Supplementary Figs. 7 and 8).

### High GAC correlates with shortened lifespan in middle-aged mice

**Fig. 4:**
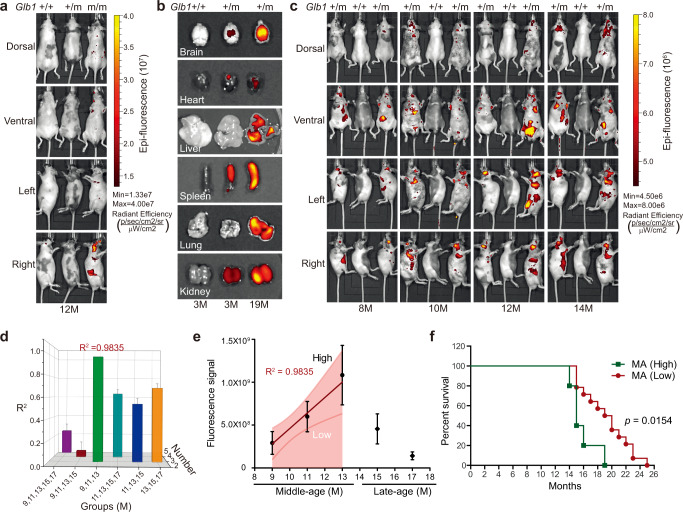
Live-imaged GAC signal correlates with lifespan. **a** Representative images showing live GAC signal detected by an IVIS Lumina II system in *Glb1*^m/m^, *Glb1*^+/m^, and *Glb1*^+/+^ mice at 12 months of age. **b** Representative images showing live GAC signal in indicated organs isolated from 3-month-old *Glb1*^+/+^ and *Glb1*^+/m^ mice and 19-month-old *Glb1*^+/m^ mice. **c** Representative live GAC signal images of a cohort of *Glb1*^+/m^ mice (*n* = 60) at indicated ages. A *Glb1*^+/+^ mouse was included as a background control. **d** A cohort of *Glb1*^+/m^ mice (*n* = 60) at 9, 11, 13, 15, and 17 months of age were live-imaged, and linear regression between GAC signal and chronological age was analyzed. There was a close correlation between GAC signal and chronological age for months 9, 11, and 13. *X*-axis, month-groups of mice; *Y*-axis, numbers of month-groups involved for analysis; *Z*-axis, *R*^2^ of linear regression; Error bar, MSE of linear regression. **e** Plot showing linear correlation between GAC signal and chronological age in middle-aged mice (MA, 9 to 13 months, *n* = 27). A 85% confidence interval was applied to define mice with GAC high signal (GACH) and mice with low GAC signal (GACL). Data represent the means ± s.e.m. **f** Comparison of lifespan between middle-aged mice with high (*n* = 5) and low (*n* = 14) GAC signal by the Log-rank (Mantel–Cox) test. “*n*” represents number of biological replicates.

We were interested in whether the GAC signal could be live-imaged to predict aging and/or lifespan. Therefore, we detected the GAC signal using an in vivo imaging system (IVIS). Age-matched wild-type mice were included as background controls. GAC signals were successfully obtained at the whole-body level as well as the individual organ level, including in the brain, heart, liver, lung, kidney, and spleen (Fig. 4a, b). Next, a cohort of 60 *Glb1*^+/m^ mice of different ages, ranging from 9 to 17 months, was examined. To facilitate analysis, the mice were divided into five groups: 9 months ± 2 weeks (*n* = 4), 11 months ± 2 weeks (*n* = 9), 13 months ± 2 weeks (*n* = 14), 15 months ± 2 weeks (*n* = 22), and 17 months ± 2 weeks (*n* = 11). Representative live fluorescence images are shown in Fig. 4c, however no obvious correlation between the GAC signal and chronological age was observed, suggesting a complex interaction between the GAC signal and chronological age. We then screened possible age periods that might show a meaningful correlation between GAC signal and chronological age. Interestingly, a linear correlation between GAC signal (mean value at specific age in months) and chronological age was observed in the MA group (9, 11, and 13 months) (Fig. 4d, *R*^2^ = 0.9835, *n* = 27). The results show that the GAC signal progressively increased with aging in MA *Glb1*^+/m^ mice but rather started to decrease in the beginning of LA stage (Fig. 4e). We next examined the GAC signal in individual tissues/organs at MA stage. Overall GAC signal was significantly increased in MA *Glb1*^+/m^ mice in comparison with EA stage (Supplementary Fig. 9a). The GAC signal was also enhanced in isolated brain, heart, liver, and kidney tissues (Supplementary Fig. 9b, c). Consistently, the mRNA levels of *Glb1* and *mCherry*, and the protein level of mCherry were upregulated in MA group, as determined by real-time PCR and IHC staining (Supplementary Fig. 9d–f). Thus, these findings suggest that the GAC signal could serve as an in vivo aging indicator in the early stage but not late stage of life in *Glb1*^+/m^ mice. We thus focused on the MA group for further investigation.

Chronological aging is a strong indicator of tissue/organ, systemic aging, and survival. Given the close correlation between GAC signal and chronological age in middle-aged mice, when chronological age does not yet predict survival, we explored whether the GAC signal serves as an early indicator of systemic aging and lifespan. We applied a 85% confidence interval and regrouped the mice into GAC high (GACH) and GAC low (GACL) according to the GAC signal (Fig. 4e). While there was an obvious correlation between body weight and chronological age, the changes in body weight were rather negligible between GACH and GACL MA mice (Supplementary Fig. 10a). More importantly, MA mice with GACH appeared to die much earlier than those with GACL (Fig. 4f, *p* = 0.0154). Thus, GAC signal can predict lifespan expectancy at the MA stage.

### The GAC signal correlates with cardiac function decline

In addition to structural deterioration, aging is marked by function deficits in virtually all organ systems, exemplified by cardiac dysfunction and cognitive decline^6^. We thus conducted a series of behavior and function tests on the mice. We found that running activity was not correlated with GAC signal or chronological age in *Glb1*^+/m^ mice (Supplementary Fig. 10b). When we subjected the mice to a water maze assay, there was almost no difference in swimming speed and escape latency between GACH and GACL *Glb1*^+/m^ mice (Supplementary Fig. 10c, d). Times across the platform (24 and 72 h), which reflect short/long-term memory, showed only subtle differences between GACH and GACL *Glb1*^+/m^ mice at MA stage (Supplementary Fig. 10e). Therefore, GAC signal is unlikely to be associated with learning ability and memory.

**Fig. 5:**
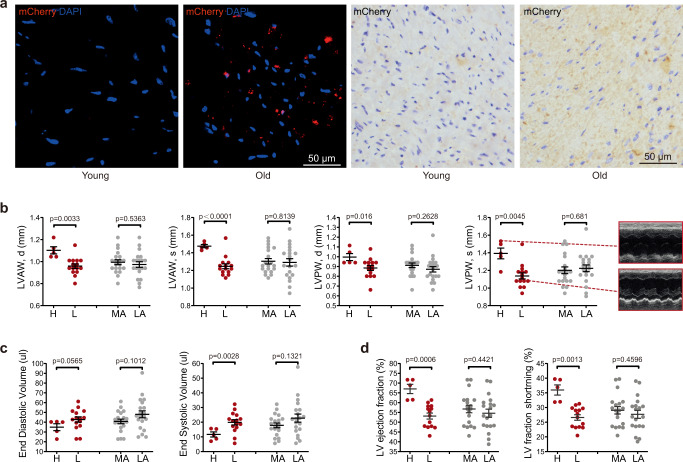
GAC signal correlates with cardiac aging and function decline. **a** Representative images showing mCherry fluorescence and anti-mCherry staining in heart sections from young (3 months, for IF and IHC) and old (23 months, for IF; 16 months, for IHC) *Glb1*^+/m^ mice. Scale bar, 50 µm. **b** Comparison of thickness of systole/diastolic left ventricle anterior wall (LVAWs/d) and systole/diastolic LV posterior wall (LVPWs/d) between *Glb1*^+/m^ mice with high (H, *n* = 5) and low (L, *n* = 15) GAC signal and between middle-aged (MA, *n* = 20) and late-aged (LA, *n* = 20) *Glb1*^+/m^ mice, as determined by echocardiography. Representative echocardiographic images of H (top) and L (middle) group mice are shown on the far right. **c** Left ventricle end diastolic volume and end systolic volume in *Glb1*^+/m^ mice grouped by GAC signal (H, *n* = 5, and L, *n* = 15) or chronological age (MA, *n* = 20, and LA, *n* = 20). **d** Left ventricle ejection fraction and fractional shortening in *Glb1*^+/m^ mice grouped by GAC signal (H, *n* = 5, and L, *n* = 14) and chronological age (MA, *n* = 19, and LA, *n* = 19). “*n*” represents number of biological replicates. Data represent the means ± s.e.m. *p* value was calculated by Student’s *t* test (two-sided).

We next examined GAC signal in heart. Fluorescence and IHC microscopy revealed GAC signal and mCherry protein levels were elevated in old *Glb1*^+/m^ mice (Fig. 5a). Further FACS analysis of isolated single cells found that percent GAC signal positive cardiac cells (ungated, total cells) was significantly increased (Supplementary Fig. 11a, 5.71% in old vs. 0.98% in young *Glb1*^+/m^ mice). GAC signal was more enriched in gated cardiomyocytes, and the percent GAC positive cardiomyocytes was increased from 1.36% in young to 12% in old *Glb1*^+/m^ mice (Supplementary Fig. 11b). We further examined cardiac function via echocardiography. For better comparisons with chronological aging, we included additional 13 mice aged 20 and 24 months. As shown, no obvious change in the thickness of systole/diastolic left ventricular anterior wall (LVAWs/d) and systole/diastolic left ventricular posterior wall (LVPWs/d), left ventricular end diastolic volume (EDV), end systolic volume (ESV), ejection fraction (EF), or fractional shortening (FS) were observed between the MA and LA groups (Fig. 5b, c). The data suggest segmental and pleiotropic effect of chronological age in cardiac structure and function. Interestingly, LVAWs/d LVPWs/d were significantly higher, whereas EDV and ESV were lower, in GACH than GACL *Glb1*^+/m^ mice at the MA stage (Fig. 5b, c). Consequently, LVEF and LVFS were significantly increased in GACH mice compared with GACL mice (Fig. 5d). Thus, the GAC signal correlates with cardiac hypertrophy and functional decline as early as middle age.

### Pathological senescence exponentially enhances GAC signal

**Fig. 6:**
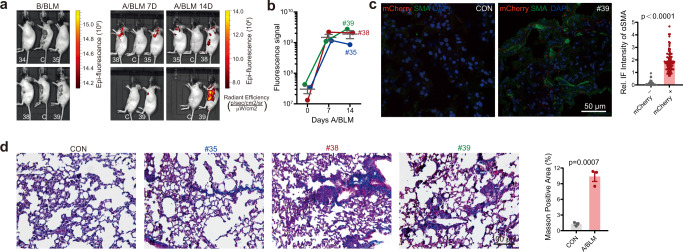
GAC signal is exponentially enhanced during pathological senescence. **a** Representative images showing whole-body GAC signal in *Glb1*^+/m^ mice before BLM treatment (B/BLM), and 7 and 14 days after BLM treatment (A/BLM 7D, A/BLM 14D). #35, #38, #39: 16 months, female, *Glb1*^+/m^ mice; C: 16 months, female, *Glb1*^+/+^ mice; #34, unrelated mice. **b** Quantification of GAC signal in **a**. *n* = 3 mice. **c** GAC signal and anti-SMA staining in lung section from *Glb1*^+/m^ mice 14 days after treatment with BLM and quantification of signal intensity. Tissues from *n* = 3 mice were quantified in each group. Over 100 cells per group were counted. Scale bar, 50 µm. **d** Masson’s trichrome staining of lung section of *Glb1*^+/m^ mice 14 days after treatment with BLM or untreated (CON) and quantification of positive area. Tissues from three mice were quantified in each group. Scale bar, 100 µm. “*n*” represents number of biological replicates. Data represent the means ± s.e.m. *p* value was calculated by Student’s *t* test (two-sided).

We next examined whether GAC signal can be used to monitor pathological aging. Treatment of mice with DNA-damaging reagent BLM induces pulmonary epithelial senescence, dramatically decreased body weight. Significantly, the whole-body GAC signal was enhanced by up to 100-fold within 1‒2 weeks after BLM treatment, peaking at around 7 days (Fig. 6a, b). This is consistent with histological data showing increased GAC signal (Fig. 6c) and fibrosis (Fig. 6d) in lung sections of BLM treated *Glb1*^+/m^ mice. Although the mRNA levels of *Glb1*, *mCherry*, *p16*^*Ink4a*^ and *p21*^*Wif1*^ were not much changed upon BLM treatment, protein levels of GLB1, p16^Ink4a^, p21^Wif1^ and mCherry were largely upregulated, as determined by RT-PCR, western blotting and IHC staining (Supplementary Fig. 12a–c). BLM treatment enhanced SAβ-gal staining in the lung (Supplementary Fig. 12d). Thus, GAC signal can be used to visualize pathological aging/senescence, as exemplified by BLM-induced lung epithelial senescence.

### Senolytic dasatinib and quercetin reduces GAC signal in BLM treated mice

**Fig. 7:**
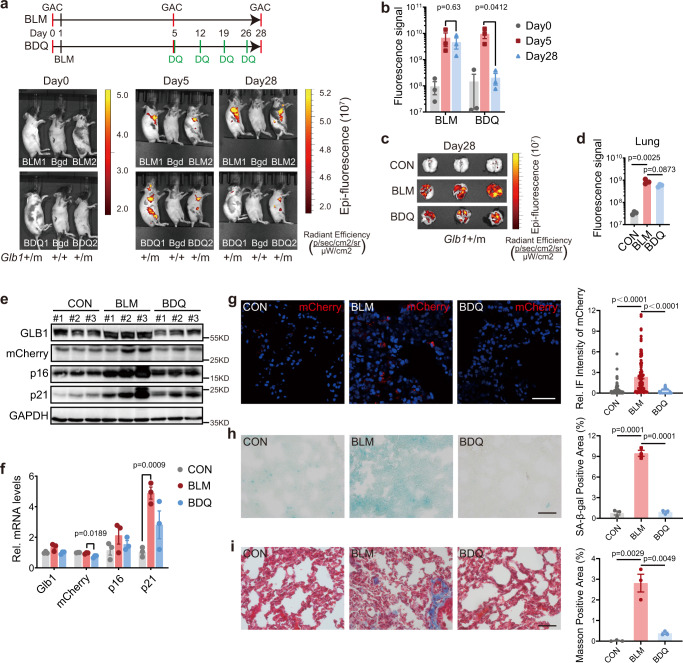
Senolytic therapies attenuate GAC signal in *Glb1*^+/m^ mice. Upper, the timeline of BLM treatment, BLM + D + Q treatment (BDQ) and GAC imaging; lower, representative images (**a**) and quantification (**b**) of the GAC signal in lung anatomical location from BLM (4 months, female, *n* = 3) and BDQ (4 months, female, *n* = 3) treated *Glb1*^+/m^ mice. 4 months female *Glb1*^+/+^ mouse was used for background (Bdg) fluorescence subtraction. Representative images (**c**) and quantification of the GAC signal (**d**) in lung tissues from CON (4 months, female, *n* = 3), BLM (4 months, female, *n* = 3) and BDQ (4 months, female, *n* = 3) *Glb1*^+/m^ mice on day 28 of the treatment in **a**. The protein (**e**) and mRNA (**f**) levels of *Glb1*, *mCherry*, *p16*^*Ink4a*^ and *p21*^*Wif1*^ in lung sections from CON, BLM, and BDQ treated *Glb1*^+/m^ mice. *n* = 3 mice. Representative images showing GAC signal (**g**), SAβ-gal staining (**h**), Masson’s trichrome staining (**i**) in lung sections from CON, BLM, and BDQ treated *Glb1*^+/m^ mice. Quantification of signal intensity and positive area is shown in right panels. Over 105 cells per group were counted in **g**. Tissues from three mice were quantified in each group. Scale bar, 50 µm. “*n*” represents number of biological replicates. Data represent the means ± s.e.m. *p* value was calculated by Student’s *t* test (two-sided).

We next investigated the senolytic treatment in GAC model. We first generated BLM-induced in vivo senescence model by intratracheal instillation of BLM in *Glb1*^+/m^ mice. We then treated the lung-injured mice with senolytic dasatinib plus quercetin (D + Q) or vehicle by intraperitoneal injection once a week for total 4 injections. The GAC signal at lung anatomical location and dissected lung tissues was increased by BLM but significantly reduced by D + Q treatment compared with vehicle-treated controls (Fig. 7a–d). Western blotting and quantitative PCR (qPCR) results showed that both protein and mRNA levels of *Glb1*, *mCherry*, *p16*^*Ink4a*^ and *p21*^*Wif1*^ were significantly increased by BLM but decreased after D + Q treatment (Fig. 7e, f). Consistently, the GAC signal was decreased in lung sections of D + Q treated group (Fig. 7g). Further, we found that both SAβ-gal-positive stain and lung fibrosis were reduced by D + Q treatment (Fig. 7h, i). Together, the results indicate that senolytic D + Q could eliminate senescence-associated markers, which is monitored by in vivo GAC signal.

## Discussion

Aging inevitably increases the risk of disease, as exemplified by CAD, AD, and cancer. Monitoring the aging process and understanding its mechanisms will not only enhance early diagnoses, it may also provide strategies for the early prevention and treatment of diseases. While biomarkers for cellular senescence in in vitro cultured mammalian cells are already well-defined, those that define in vivo senescence/aging at the systemic level remain scarce. Here, we generated a targeted *Glb1*^*+/*m^ allele at the *Glb1* locus that encodes β-galactosidase. The GAC signal indicates *Glb1* level. The results reveal that the live-imaged GAC signal is linearly correlated with chronological age, but only in middle-aged mice (9–13 months). High GAC at the MA stage was associated with cardiac hypertrophy and shortened lifespan. Moreover, GAC signal was exponentially increased in pathological lung fibrosis induced by BLM. Thus, this in vivo reporter mouse can faithfully monitor systemic aging and organ functional decline in a manner closely associated with lifespan, and provides an ideal system for studying aging mechanisms and developing anti-aging manipulations.

The upregulation of *p16*^*Ink4a*^ transcription and elevated SAβ-gal staining are both well-established and widely used biomarkers for cellular senescence^17^, and the former led to the generation of live-imaging aging reporter mice^20,22,23^. Intriguingly, high level of *p16*^*Ink4a*^, indicated by luciferase activity, predicts cancer initiation rather than lifespan. Similarly, the in vivo application of SAβ-gal as a senescence marker at the tissue level is also limited. Positive SAβ-gal-staining is easy to obtain in kidney and adipose tissue sections but difficult to obtain in blood vessel and heart sections. By SAβ-gal staining, not many positive cells were detected in old individuals^26^. It raises the question of whether SAβ-gal labels in vivo senescence or if the percentage of in vivo senescent cells is indeed very low. It cannot be explained by cell rejuvenation hypothesis which argues that the senescent cells are replaced by newly differentiated cells from adult stem cells, because at the very end when stem cell pool is depleted, the senescent cells will ultimately accumulate. Another possibility is that the senescent cells are simply removed and the individual dies of degeneration, which is an important hallmark of systemic aging. Organ function decline represents another hallmark of systemic aging. The SAβ-gal staining reflects the activity and level of β-D-galactosidase encoded by *Glb1*^25^. Thus, we thought that coupling *Glb1* transcription to an mCherry signal might avoid the subjective effects of SAβ-gal staining^27^ and facilitate live-imaging analyses. Interestingly, the high GAC signal in MA mice points to cardiac hypertrophy, which might be one reason for the earlier mortality of GACH mice. Thus, the GAC signal may serve as an early indicator of heart dysfunction. Cardiomyocytes lose proliferation capacity in adulthood and if any, undergo SIS rather than replicative senescence. This might explain why a SAβ-gal signal is easily detected in pathological senescence, as it is indeed SIS. Of note, at tissue level, SAβ-gal can be easily detected in endothelial and epithelial injuries induced by angiotensin II, BLM, and DOX^21,28,29^. Contrastingly, the p16^Ink4a^ reporter is preferentially linked to malignancy^22^. Therefore, it is plausible that the two reporters may preferentially indicate different types of in vivo senescence, i.e., SIS by GAC reporter in post-mitotic tissues and replicative senescence by p16^Ink4a^ reporter in proliferating tissues.

Although GAC is linearly and positively correlated with chronological age in middle-aged mice, such correlation is diminished in late-aged mice (Fig. 4d, e). Interestingly, a recent transcriptomic study of murine aging also revealed a transition period at around 10 months of age associated with changes in pathways that regulate protein folding and stress response^30^. A study of plasma protein from 18–95 years old population revealed significant changes in signaling pathways in their 40s^31^. Similarly, peripheral blood mononuclear cells underwent obvious epigenomic changes around 40 years in healthy population^32^. Therefore, we reason there might be a MA to LA transition stage and the potential underling mechanism needs further investigation. Cardiac hypertrophy is an adaptive response to environmental stimuli and may cause sudden death or progress to heart failure^33^. We speculated that cardiac dysfunction might be behind the early death of the GACH mice. Notably, no difference was observed between MA and LA mice in terms of heart phenotypes and large variations was observed in individual mouse in the examined parameters, supporting the notion that mice are poor models of spontaneous cardiac disease. In other words, owing to the heterogeneity of mice aging population, chronological age alone may not be a robust risk factor of heart function, e.g., cardiac hypertrophy. Indeed, the most significant finding in our study is that we could separate about one-fifth of a total population by high GAC signal at MA stage, and this small population of GACH mice developed cardiac hypertrophy. Therefore, the live GAC signal could serve as an indicator of biological aging instead of chronological aging. Supporting the hypothesis, dramatic increase of GAC signal and hypertrophic heart as an adaptive response to environmental stimuli was observed, which is independent of chronological age. High GAC signal points to cardiac hypertrophy in MA mice, which might lead to early death. By contrast, mice with low GAC may survive through this period. In this regard, the diminished GAC signal in late-aged mice might attribute to such selection effect, i.e., those mice that have low GAC dominate the LA population. Of note, while the medium lifespan of experimental C57BL/6 mice from individual laboratories varies from 18 to 25 months^34–37^, in our current study, it is about 19 months (Fig. 4f). Such short-lived variation might be attributable to a side-effect of experimental manipulation, like GAC signal imaging (including overnight fasting, anesthesia, whole-body shaving and imaging), echocardiographic evaluation after anesthesia and 8 days of Morris water maze assay.

Despite advances in aging research, fundamental questions remain. The GAC reporter mice together with single-cell transcriptomics could help dissect the senescence/aging heterogeneity and facilitate anti-aging interventions. We showed that the GAC signal can help monitor the senolytic effect of D + Q in pathological senescence model. This model is particularly useful for studies of tissue/organ aging and pathologies, such as cardiac dysfunction, AD, tissue fibrosis, metabolic disorders, and wound healing. From a translational perspective, it would be worthwhile to develop chemical probes that can convert β-galactosidase activity into signals to visualize the in vivo aging process. In summary, the GAC reporter mice can monitor systemic aging and functional decline and predict lifespan, which make it an ideal model that may facilitate the study of aging mechanisms, and the development of strategies for the early diagnosis, prevention, and treatment of aging and aging-related diseases.

## Methods

### Mouse models

The *Glb1-2A-mCherry* knock-in allele (*Glb1*^m^) in C57BL6/J background was generated using the CRISPR/Cas9 system by Shanghai Biomodel Organism Science & Technology Development Co. Ltd. (Shanghai, China). Briefly, the coding sequences of 2A peptide and mCherry were sequentially added to the 3′ end of the *Glb1* open reading frame (ORF), thus generating a new ORF encoding a Glb1-2A-mCherry fused peptide. The “self-cleaving” property of the 2A peptide was expected to generate a Glb1 peptide and a mCherry peptide. All mice were housed at 21–23 °C, 40–60% humidity, and with a 12 h light/12 h dark light cycle under specific pathogen-free conditions. In accordance of the laboratory animal welfare and ethical, the raising density (no more than 5 adult mice in an IVC cage) is rigidly controlled, and the cages including the food and water are refreshed weekly. The humane endpoint for euthanasia are set as follows: rapid weight loss of ≥20% of body weight; no response to gentle stimulation; difficulty breathing; inability/unwillingness to ambulate to reach food or water. Animal experiments were conducted in accordance with ethical and scientific protocols approved by the Committee on the Use of Live Animals in Teaching and Research of Shenzhen University, China.

### GAC signal imaging (mCherry detection)

GAC signal in *Glb1*^+/m^ mice was imaged using an IVIS Lumina II system (Caliper Life Sciences, USA). A wide-angle lens was used to simultaneously capture images from >3 animals. Wild-type C57BL6/J littermates from heterozygous crosses were used as the control for background signal subtraction. Images were obtained using a binning of 4 (medium), F/Stop of 2, excitation filter of 535 nm, and DsRed emission filter. Living Image Software (Caliper Life Sciences, USA) was used to compare multiple images taken at the same exposure time. For all experiments, mice were randomly grouped, but ensuring that was age- and gender-matched.

### Morris water maze

*Glb1*^+/m^ mice were trained in a round water-filled tub (160 cm in diameter and 50 cm in height). Each mouse was given four trials per day for 5 consecutive days with a 15-min inter-trial interval. The first day was designated the training day 1, and the maximum trial length was 60 s. If mice did not reach the platform in the allotted time, they were manually guided to it, where they remained for 15 s. After the 5 days of task acquisition, probe trials were performed 24 and 72 h later to assess the mice’s short-term and long-term memory consolidation. The platform was removed, and the mice were placed into a quadrant of the pool opposite to the original platform quadrant. In each probe trial, mice were allowed to swim for 60 s. The time mice spent in the original platform quadrant and the number of times the mice crossed the platform position were recorded. All trials were recorded using an HVS water maze program for subsequent analyses of escape latency and swimming speed (WaterMaze3, Actimetrics, USA).

### Echocardiographic evaluation

*Glb1*^+/m^ mice were anesthetized by 1.5–2% isoflurane gas inhalation, then subjected to transthoracic echocardiography (Vevo 2100 Imaging System, VisualSonics, USA). Heart rate, cardiac output, LVEF, LVFS, EDV, ESV, LVAWs/d and LVPWs/d were recorded.

### Bleomycin-induced pulmonary epithelial senescence

Three 16-month-old *Glb1*^+/m^ mice received intratracheal instillation of bleomycin (HY-17565, MCE, USA) in PBS at a dose of 3 mg/kg. Overall GAC signals were measured with the IVIS Lumina II system 7 and 14 days after the treatment. Mice were sacrificed on day 14, and lung samples were collected for further analysis.

### Dasatinib plus quercetin (D + Q) treatment

Nine *Glb1*^+/m^ mice were divided into three groups: vehicle treated (CON), bleomycin treated (BLM), and BLM and D + Q treated (BDQ). GAC signal was imaged using an IVIS Lumina II system (Caliper Life Sciences, USA). Group BLM and BDQ received intratracheal instillation of bleomycin in PBS (Nippon Kayaku) at a dose of 1.5 mg/kg on Day 0. On Day 5, dasatinib (1 mg/kg, S1021, Selleck, USA) plus quercetin (10 mg/kg, S2391, Selleck, USA) mixed in 90% PBS, 5% Tween-80, and 5% polyethylene glycol (PEG) was administrated to Group BDQ mice by intraperitoneal (i.p.) injection, once a week for 4 weeks. Mice were sacrificed on Day 28, and lung samples were collected for further analysis.

### Hematoxylin-eosin, immunohistochemical, and Masson’s trichrome staining

Hematoxylin-eosin (H&E) and IHC staining were performed as described previously^38^. Briefly, for H&E staining, paraffin-embedded sections of paraformaldehyde (PFA)-fixed tissues were dewaxed and rehydrated, then processed in the order of hematoxylin staining, differentiation, bluing, dehydration, eosin staining, clearing, and addition of cover-slip. For IHC staining, the 3,3′-diaminobenzidine (DAB) staining method was used. Endogenous peroxidases were blocked using blocking solution (PV-6001, ZSGB-BIO, China). After blocking with serum, the primary antibody and biotinylated secondary antibody incubation steps, and addition of streptavidin-HRP, the DAB substrate (8059, Cell Signaling Technology, USA) was used to develop the signal color. Masson trichrome staining was performed using the kit from Solarbio (G1340, Solarbio, China) following the manufacturer’s instructions.

### Cell culture

*Glb1*^+/m^ and wild-type MEF cells were isolated from embryos on day 13.5 of gestation. Primary normal human fibroblasts (HuFB) were isolated from the skin tissue of a healthy female donor^27^ and the written informed consent was obtained from the donor. The use of human fibroblasts was approved by the Medical Ethic Committee of Shenzhen University. Cells were maintained in DMEM with FBS (PAN SERATECH, Germany) (10% for MEF and 15% for human fibroblast), GlutaMAX (35050061, Gibco, Thermo Fisher, USA), non-essential amino acids (11140050, Gibco, Thermo Fisher, USA), penicillin and streptomycin (Gibco). All cells were passaged every 3 days and cultured at 37 °C in a humidified incubator with 5% CO_2._

### Stress induced cellular senescence

For the doxorubicin (DOX) or bleomycin (BLM) induced senescence, MEF cells were treated with 200 nM of DOX for 24 h or 6 μg/mL of BLM for 48 h. For IR induced senescence, MEF and HuFB fibroblasts were exposed to 10 Gy of IR in an X-ray Biological Irradiator (RS2000, Rad Source Technologies). For H_2_O_2_-induced senescence, MEF and HuFB cells were treated with freshly prepared 400 μM of H_2_O_2_ for 4 h, and 200 μM of H_2_O_2_ for another 4 h on next day. For oncogene-induced senescence, HuFB cells were infected with the lenti-virus system expressing the activated mutant hRAS (G12V). On day 12 after treatment, cells were harvested for further analysis.

### Senescence-associated β-galactosidase staining

Frozen tissue sections and in vitro cultured MEF cells were prepared. SAβ-gal staining was performed with the senescence β-galactosidase staining kit (9860, Cell Signaling Technology, USA) according to the manufacturer’s protocol. The tissue sections were incubated in staining buffer in a dry incubator at 37 °C for 20–24 h to develop the color, and MEF cells were incubated for 16 h. For the analysis of the co-localization of mCherry fluorescence and β-galactosidase staining in tissue, the sections were fixed in 1% PFA for 10 min and the performed regular staining.

### SAβ-galactosidase activity analysis by C12FDG staining

To examine the β-galactosidase (β-gal) activity by flow cytometry, MEF cells were incubated with 20 μM of C12FDG (C131100, Aladdin, China) for 0.5–1 h and HuFB cells were incubated with 33 μM of C12FDG for 1.5–2 h. Cells were washed with PBS and harvested for flow cytometry analysis. Forward scatter area (FSC-A) and side scatter area (SSC-A) was used to sort for cells, forward scatter area (FSC-A) and forward scatter height (FSC-H) was used to sort for single cells, and FITC channel was used to sort for C12FDG-positive cells in SIS cells.The data were analyzed using the FlowJo software (FlowJo LLC, USA).

### FACS analysis of GAC signal in cardiac cells and cardiomyocytes

After mice were euthanized, the hearts were perfused with pre-cold perfusion buffer and then anterior wall of left ventricle was removed to digestion buffer in 37 °C shaking water bath for 15 min. After digestion of cardiac tissue, the cell lysate passed through a 100 µm cell strainer and cells we collected were resuspended in DMEM with DAPI (D3571, Invitrogen) for flow cytometry analysis by FACS Aria II (BD bioscience). Forward scatter area (FSC-A) and side scatter area (SSC-A) was used to sort for cells, DAPI negative was used to sort for living cells, and PE channel was used to sort for mCherry-positive cells in cardiac cells. For sorting mCherry-positive cardiomyocytes, we employed additional FITC channel to sort for ~30% cardiomyocytes in cardiac cells with green autofluorescence before PE channel selection.

### Immunofluorescence staining

MEF cells and frozen tissue sections were fixed in 4% PFA for 5 min and permeabilized with 0.2% Triton X-100 for 5 min. After blocking with 3% BSA for 1 h, samples were incubated with primary antibodies overnight at 4 °C and, subsequently, incubated with fluorescence-conjugated secondary antibodies for another 1 h at room temperature. After staining the nucleus with DAPI, the samples were mounted in anti-fade medium and covered with a glass coverslip. Images were collected using the DragonFly confocal imaging system (Andor).

### Western blotting

Cells and tissues were lysed in RIPA buffer (20 mM Tris-HCl pH 7.5, 150 mM NaCl, 1 mM EDTA, 1 mM EGTA, 1% NP40, 1% Triton X-100, 1% Na-deoxycholate, 0.1% SDS, 1 mM PMSF, and protease inhibitor cocktail), and protein was quantified using the PierceTM BCA kit (23227, Thermo Fisher, USA). Protein samples were subjected to SDS-PAGE electrophoresis and transferred to a PVDF membrane. After blocking with 5% fat-free milk in TBS-T buffer (TBS buffer, 10 mM Tris-HCl pH 7.5, 150 mM NaCl, with 0.1% Tween-20), the membrane was incubated with relative primary antibodies overnight at 4 °C. Subsequently the secondary antibodies conjugated HRP were added and incubated for 1 h at room temperature before detection using ECL substrate solution (34578, Thermo Fisher, USA) by Image Lab Software (Bio-Rad, V5.2.1 build 11). ImageJ software (V1.52) was used for quantification analysis. All antibodies are listed in Supplementary Table 1.

### RNA-extraction and q-RT-PCR

Cell and tissues were lysed in TRIzol reagent RNAiso Plus (9109, Takara, Japan), and total RNA was extracted according to the manufacturer’s instructions. RNA quality and concentration were analyzed using NanoDrop One (Thermo Fisher, USA). A total of 2 μg of RNA from each sample was used for cDNA synthesis, and qPCR was performed on the CFX Connect Real-Time System (Bio-Rad) using Hieff qPCR SYBR Green Master Mix (11201, Yeasen, China). Relative gene expression was quantified by normalizing to *Gapdh* values. Primers for RT-PCR are listed in Supplementary Table 2.

### Quantitative analyses of imaging data

Quantification of images obtained from immunofluorescence, immunohistochemistry (IHC), Masson’s trichrome and SAβ-gal staining were performed with the ImageJ® software. For Immunofluorescence and Masson’s trichrome staining, the RGB form of images were converted into 8-bit gray and RGB-stack form respectively. The region of interest (ROI) were determined by adjusting intensity threshold, and the positive signals were measured and presented as signal intensity or positive area. For IHC, the IHC-toolbox was adopted and the ROI was determined by using the training function. The RGB images were converted into 8-bit gray form and the positive staining area of the ROI was measured after adjusting intensity threshold. The SAβ-gal tissue staining was quantified as Masson’s trichrome staining. For cellular SAβ-gal staining, the nuclei were counterstained with DAPI for accurate and convenient counting of cell number, and percent SAβ-gal-positive cells was presented. All parameters were kept consistent for each experiment during the data analyses.

### Statistical analyses

Statistical analyses were performed with Excel (Microsoft 365 Family) and GraphPad Prism 9.2.0 software (GraphPad Software Inc., USA). The Log-rank (Mantel–Cox) test were used to analyze survival rate, linear regression was used to calculate the coefficient of determination (*R*^*2*^) and mean squared error, 85% confidence interval was applied to determine GACH and GACL groups, and all other data were analyzed using unpaired Student’s *t* tests (two-sided). All experiments were performed with at least three biological replicates. The data are presented as the mean ± s.e.m. *p* < 0.05 was considered to indicate a statistically significant difference.

### Reporting summary

Further information on research design is available in the Nature Portfolio Reporting Summary linked to this article.

## Supplementary information

Supplementary information

Reporting Summary

## Data Availability

All data generated or analyzed during this study are included in this published article (and its Supplementary Information files). Source data are provided with this paper.

## Source data

Source Data
