# Supplementary material for: A Glb1-2A-mCherry reporter monitors systemic aging and predicts lifespan in middle-aged mice
Source: Nat Commun. 2022 Nov 17;13:7028. doi: 10.1038/s41467-022-34801-9 (PMC9671911; doi:10.1038/s41467-022-34801-9)

Supplementary information

This file contains Supplementary Figures 1 to 12 with figure legends, Supplementary Tables 1 to 2, and uncropped blot images for the supplementary figures.

A Glb1-2A-mCherry reporter monitors systemic aging and predicts lifespan in middle-aged mice

Jie Sun[1,2,3,#], Ming Wang[1,2,#], Yaqi Zhong[1,#], Xuan Ma[1], Shimin Sun[1], Chenzhong Xu[1,2], Linyuan Peng[1], Guo Li[4], Liting Zhang[1], Zuojun Liu[1,2], Ding Ai[5], Baohua Liu[1,2,3*]

[1]Shenzhen Key Laboratory for Systemic Aging and Intervention (SKL-SAI), School of Basic Medical Sciences, Shenzhen University, Shenzhen 518055, China

[2]Guangdong Key Laboratory of Genome Stability and Human Disease Prevention, Marshall Laboratory of Biomedical Engineering, National Engineering Research Center for Biotechnology (Shenzhen), International Cancer Center, Shenzhen University, Shenzhen 518055, China

[3]Shenzhen Bay Laboratory, Shenzhen, China

[4]Department of Dermatology, Xiangya Hospital, Central South University, Changsha, China

[5]Department of Physiology and Pathophysiology, Tianjin Medical University, Tianjin, 300070, China

[#]These authors contributed equally.

*Correspondence should be addressed to Dr Baohua Liu (ppliew@szu.edu.cn)

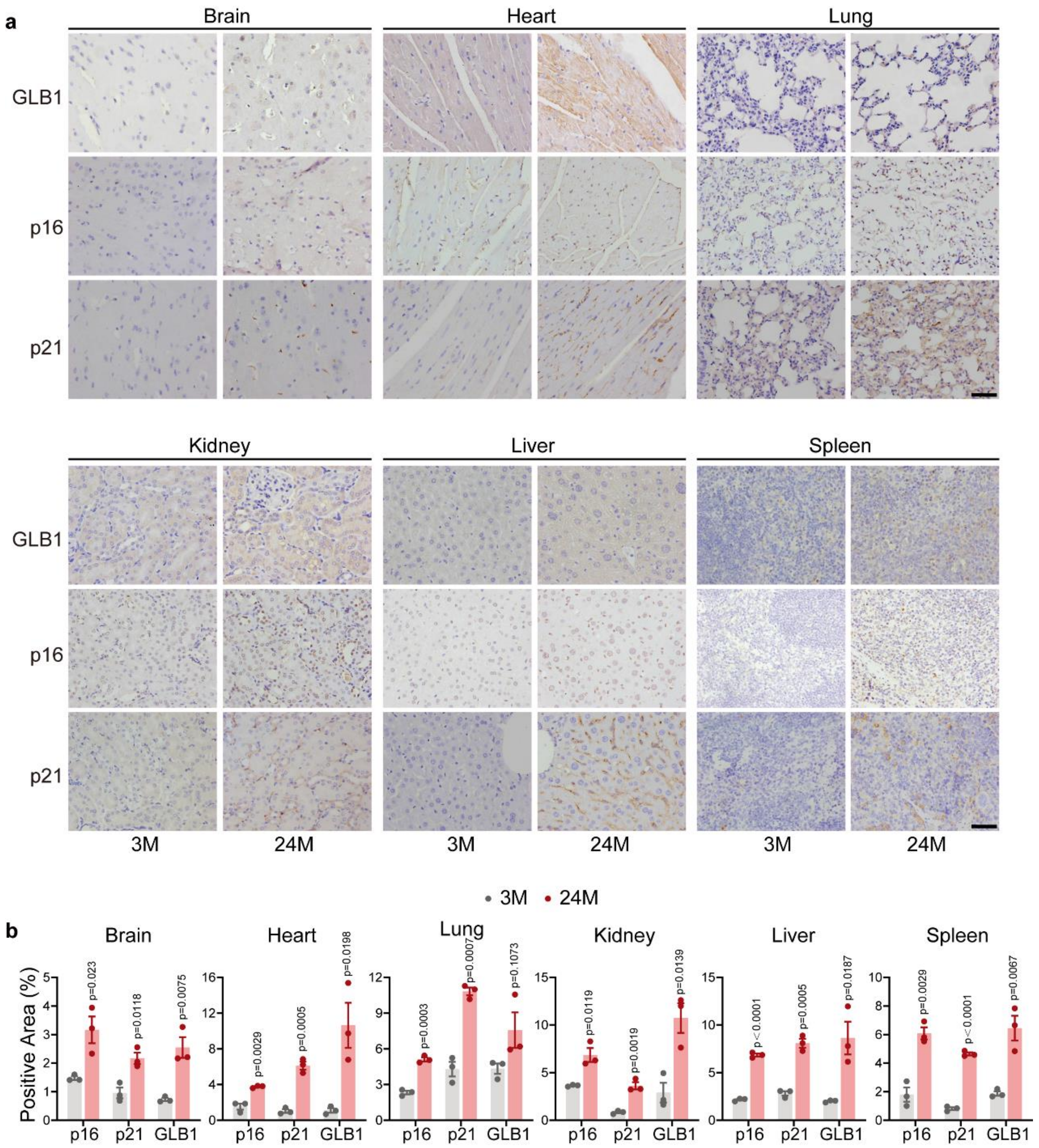

Supplementary Figure 1 Histochemical analysis of senescence markers in murine tissues

a Immunohistochemical staining of GLB1, $p16^{Ink4a}$ and $p21^{Wif1}$ in indicated tissues isolated from young (3 m, male, n = 3) and old (24 m, male, n = 3) mice. Scale bar, 50 µm. **b** Quantification of (**a**). ‘n’ represents number of biological replicates. Data represent the means ± s.e.m. *P* value was calculated by Student’s *t*-test (two-sided).

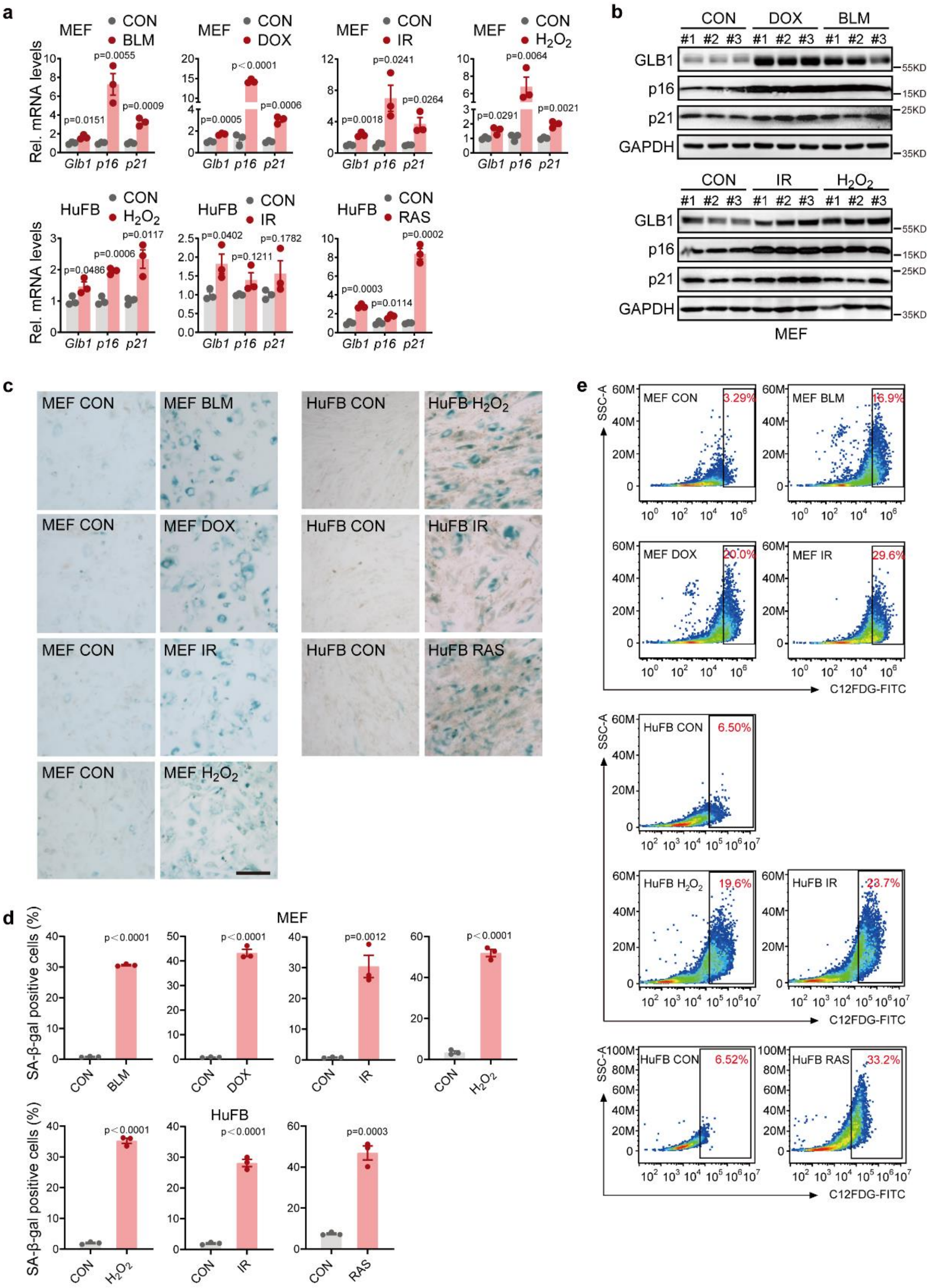

Supplementary Figure 2 Increased *Glb1* levels in stress-induced senescence

a-e The mRNA levels (**a**, cells from n = 3 embryos) and protein levels (**b**) of *Glb1*, *p16*^{Ink4a} and *p21*^{Wif1}, SAβ-gal staining and quantification of positive cells (**c, d**, over 300 cells were counted in each group), and percentage of C12FDG-positive cells (**e**) in passage 2 (P2) MEFs and passage 21 (P21) human fibroblast (HuFB) during stress-induced senescence. Scale bar, 100 µm. Cell passage (P) numbers are indicated. ‘n’ represents number of biological replicates. Data represent the means ± s.e.m. *P* value was calculated by Student’s t-test (two-sided).

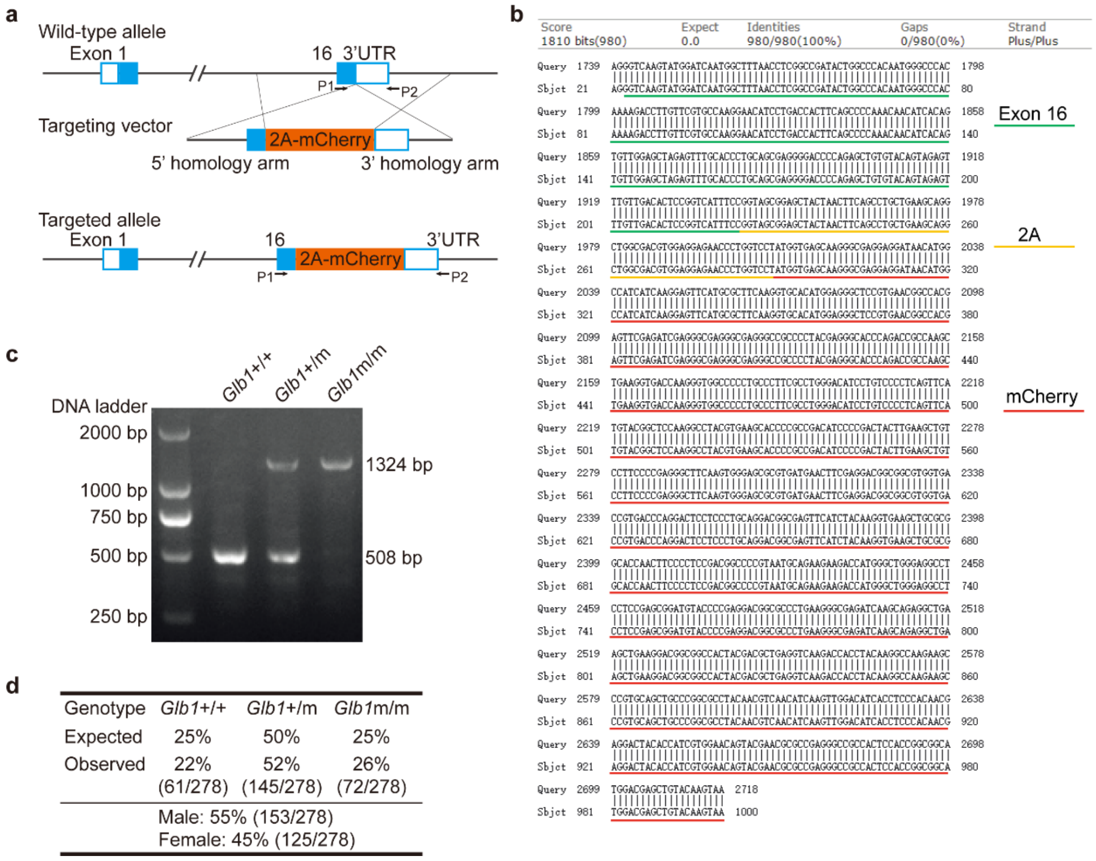

Supplementary Figure 3 Characterization of GAC allele

a Schematic of gene-targeting strategy of *Glb1*$^{+/m}$ allele–Glb1-2A-mCherry reporter. The crossed lines indicate homogenous recombination site. P1, P2, primers for targeting PCR verification. **b** Genome sequencing data showing the correct and sequential insertion of 2A-coding sequence and mCherry-coding sequence in exon 16 of *Glb1* gene. **c** Target-specific PCR amplification of wild-type and GAC allele in *Glb1*$^{+/+}$, *Glb1*$^{+/m}$, and *Glb1*$^{m/m}$ mice using primer P1 and P2 as indicated in (**a**). A 508-bp band was specifically amplified from the wild-type allele and a 1324-bp band was specific to the GAC allele. **d** The birth ratio analysis of three genotypes. Noted that *Glb1*$^{+/+}$, *Glb1*$^{+/m}$, and *Glb1*$^{m/m}$ mice were born at the expected Mendel's ratio.

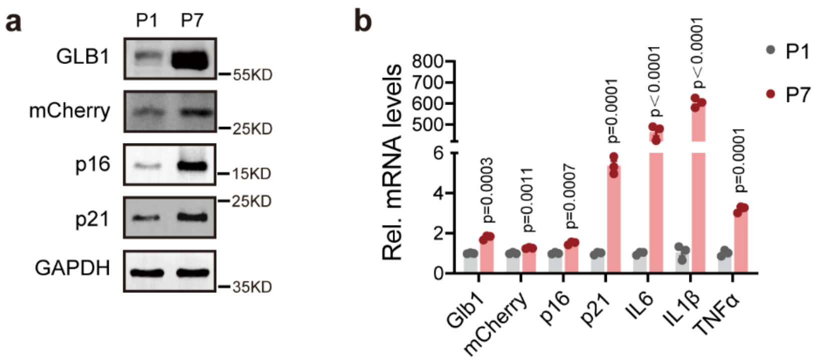

Supplementary Figure 4 Validation of MEFs from GAC mice *in vitro*

a, b Immunoblotting (**a**) and qPCR (**b**) analyses of *Glb1*$^{+/m}$ MEFs (from n = 3 embryos) underwent replicative senescence at P7. Cell passage (P) numbers are indicated. Data represent the means ± s.e.m. *P* value was calculated by Student's *t*-test (two-sided).

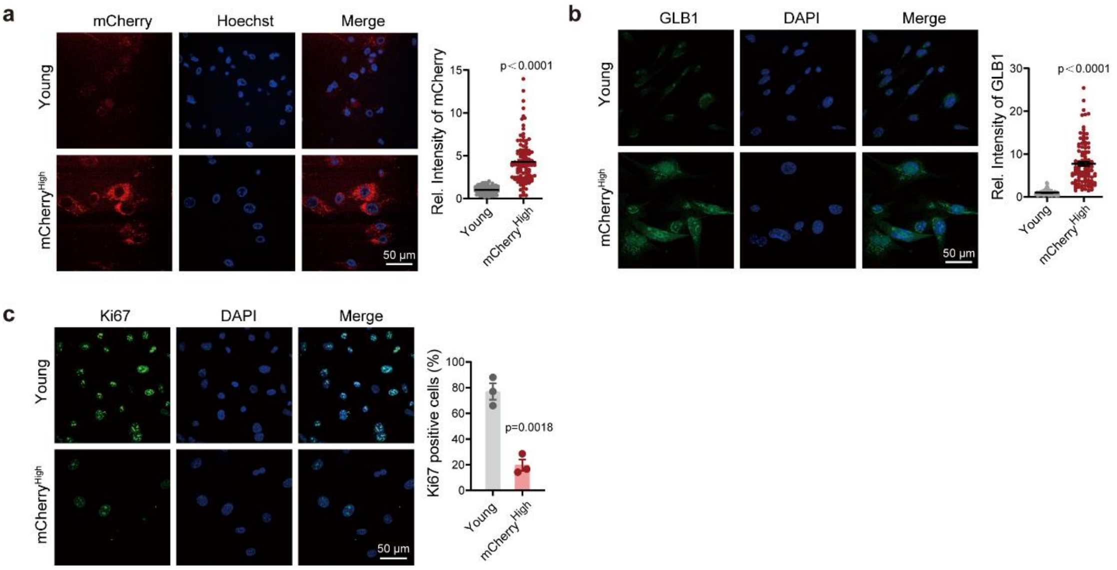

Supplementary Figure 5 Validation of FACS-sorted MEFs from GAC mice *in vitro*

a Live imaging of mCherry signals in Young (P1) *Glb1*$^{+/m}$ MEFs and FACS-sorted P7 mCherry^{High} *Glb1*$^{+/m}$ MEFs. Over 100 cells per group were counted. **b, c** Immunostaining of Young and mCherry^{High} MEFs using the indicated antibodies. Over 100 cells per group were counted in (**b**). Data represent the means ± s.e.m. *P* value was calculated by Student's *t*-test (two-sided).

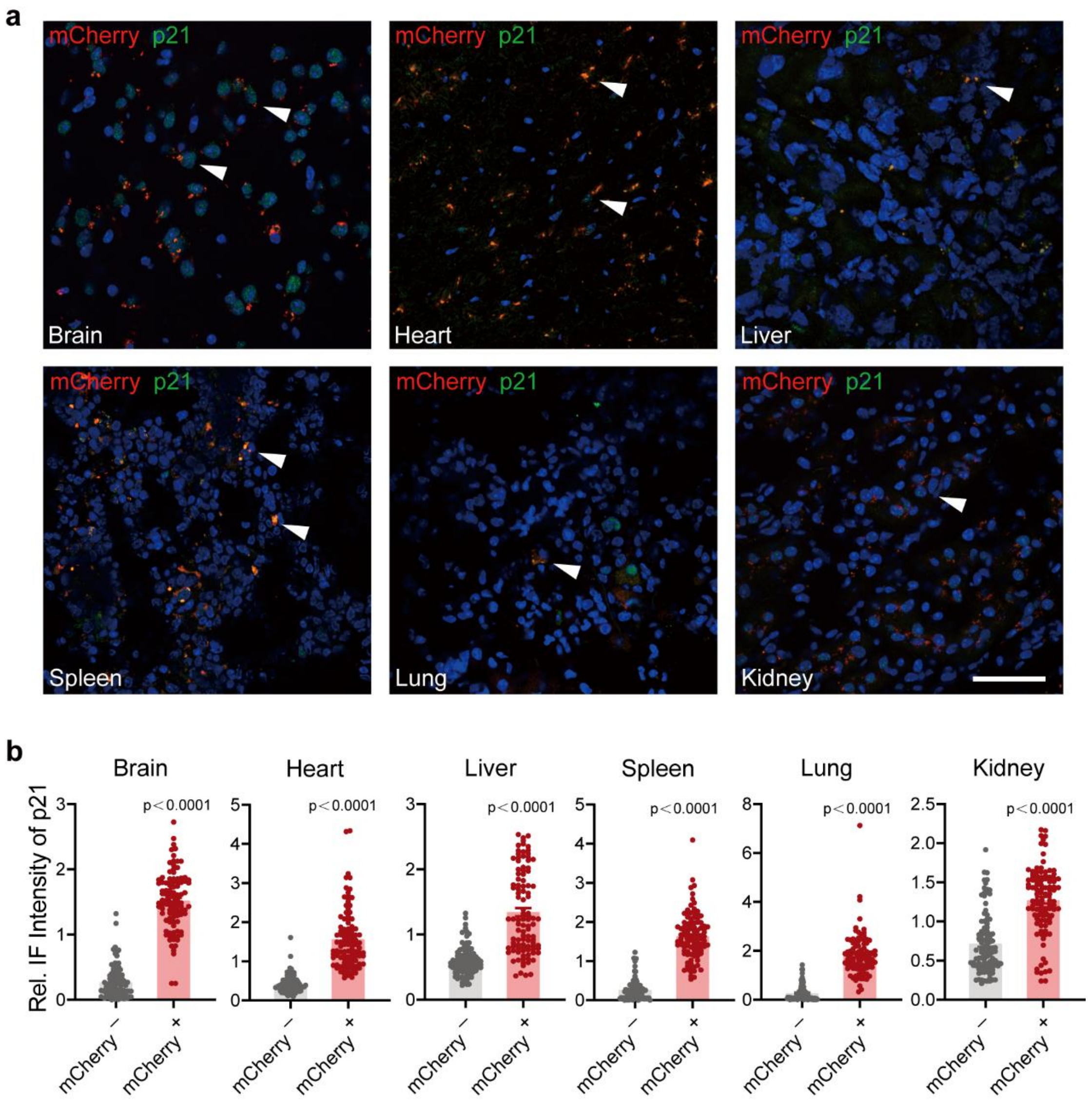

Supplementary Figure 6 Co-existence of mCherry signal and p21^{Wif1} in tissues of *Glb1*$^{+/m}$ mice

a Representative images showing co-existence of mCherry fluorescent signal (red) and anti-p21^{Wif1} staining (green) in brain, heart, liver, spleen, lung, and kidney tissues of *Glb1*$^{+/m}$ mice (24 m, male, n = 3). Arrows indicate cells with intense senescent signal. Scale bar, 50 µm. **b** Quantification of the fluorescence signal of mCherry and anti-p21^{Wif1} staining in (a). Over 100 cells per group were counted. 'n' represents number of biological replicates. Data represent the means ± s.e.m. *P* value was calculated by Student's *t*-test (two-sided).

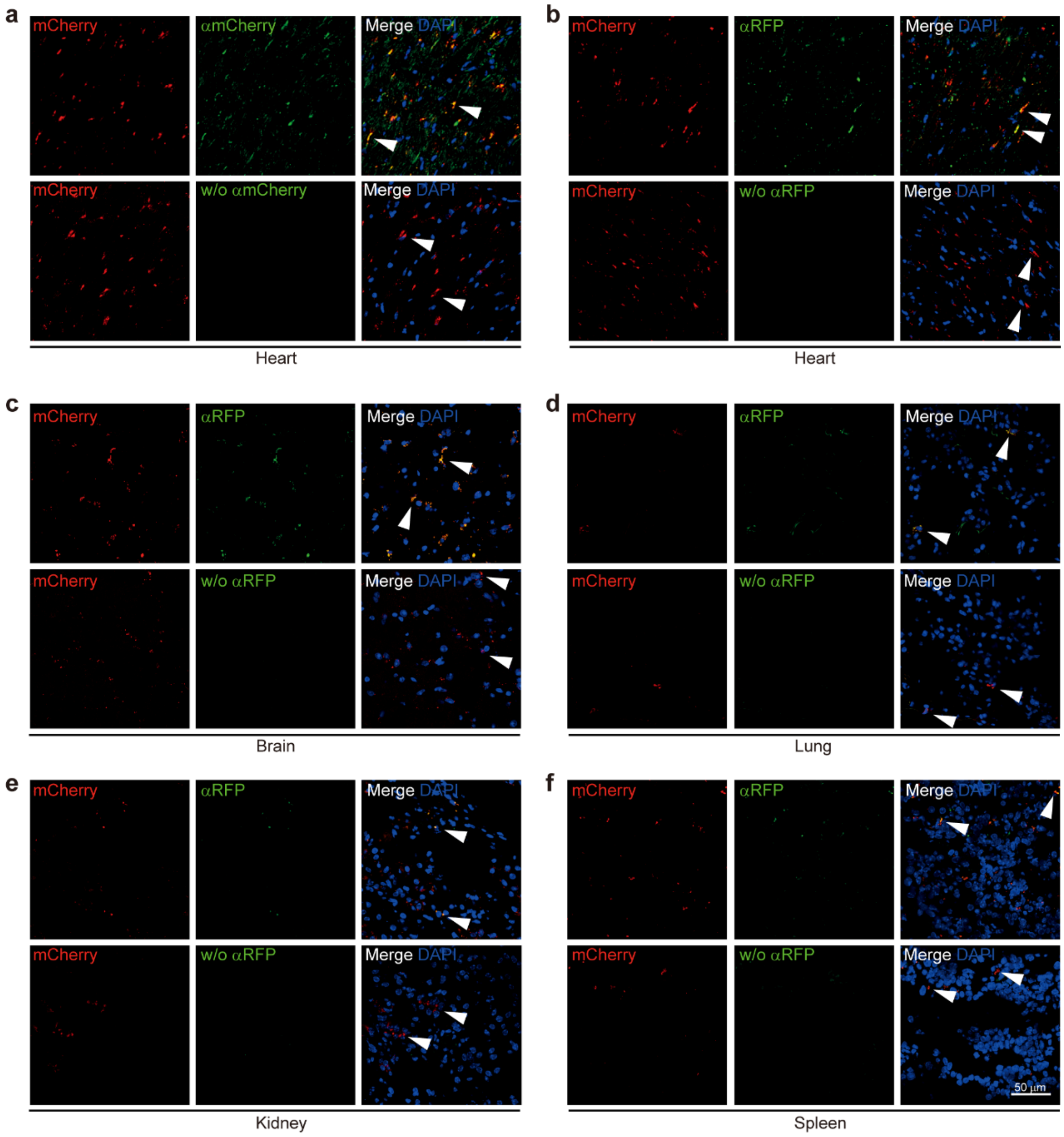

Supplementary Figure 7 mCherry signal specificity confirmed by immunofluorescence staining

a, b Representative images showing colocalization of mCherry fluorescent signal and anti-mCherry (**a**) or anti-red fluorescent protein (RFP) (**b**) staining in heart tissue from *Glb1*$^{+/m}$ mice (23 m, female, n = 3). **c-f** Representative images showing colocalization of mCherry fluorescent signal and anti-RFP staining in brain, lung, kidney, and spleen from *Glb1*$^{+/m}$ mice (23 m, female, n = 3). ‘n’ represents number of biological replicates. Arrows indicate representative cells with red and green signal colocalization (upper) and cells with only red signal in no antibody control (lower). ‘w/o’, without. Scale bar, 50 µm.

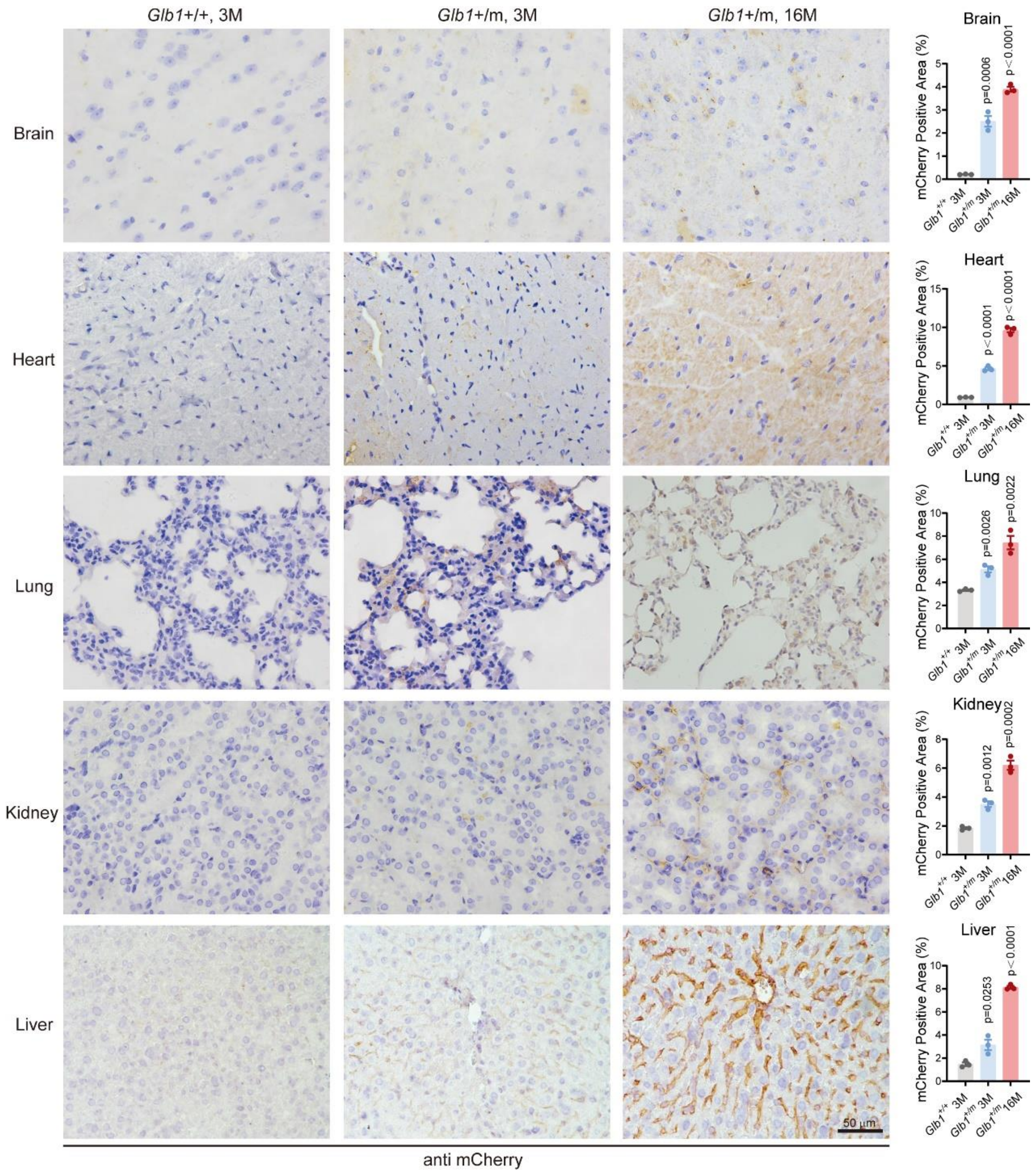

Supplementary Figure 8 Immunochemical staining of mCherry in *Glb1*$^{+/m}$ tissues

Left, Immunochemical staining of mCherry in indicated tissues of *Glb1*$^{+/m}$ (3 m, male, n = 3; 16 m, male, n = 3) and wild-type *Glb1*$^{+/+}$ (3 m, male, n = 3) mice. Right, quantification of relative staining intensity. 'n' represents number of biological replicates. Scale bar, 50 µm. Data represent the means ± s.e.m. *P* value was calculated by Student's *t*-test (two-sided).

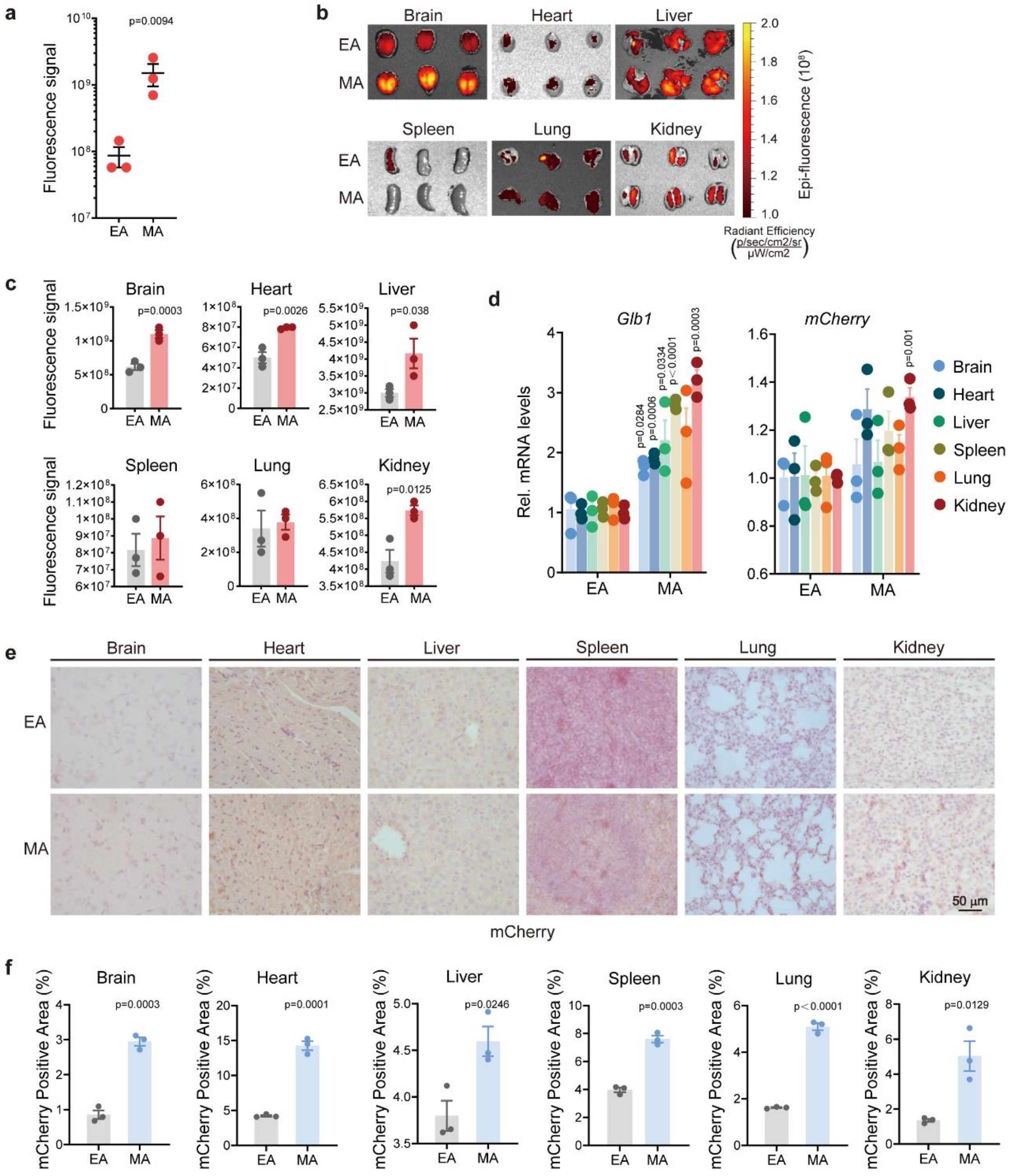

Supplementary Figure 9 GAC signal and mCherry expression in EA and MA mice

a Quantification of whole-body GAC signal from EA (3 m, female, n = 3) and MA (12 m, female, n = 3) *Glb1*$^{+/m}$ mice. **b** Representative images showing GAC signal in indicated organs isolated from EA and MA *Glb1*$^{+/m}$ mice. **c** Quantification of GAC signal in (**b**), n = 3 mice. **d** The mRNA levels of *Glb1* and *mCherry* in tissues isolated from EA and MA *Glb1*$^{+/m}$ mice (n = 3). **e** Immunochemical staining of mCherry in indicated tissues from EA and MA *Glb1*$^{+/m}$ mice. Scale bar, 50 μm. **f** Quantification of anti-mCherry staining positive area in (e). Tissues from 3 mice were analyzed in each group. ‘n’ represents number of biological replicates. Data represent the means ± s.e.m. *P* value was calculated by Student’s *t*-test (two-sided).

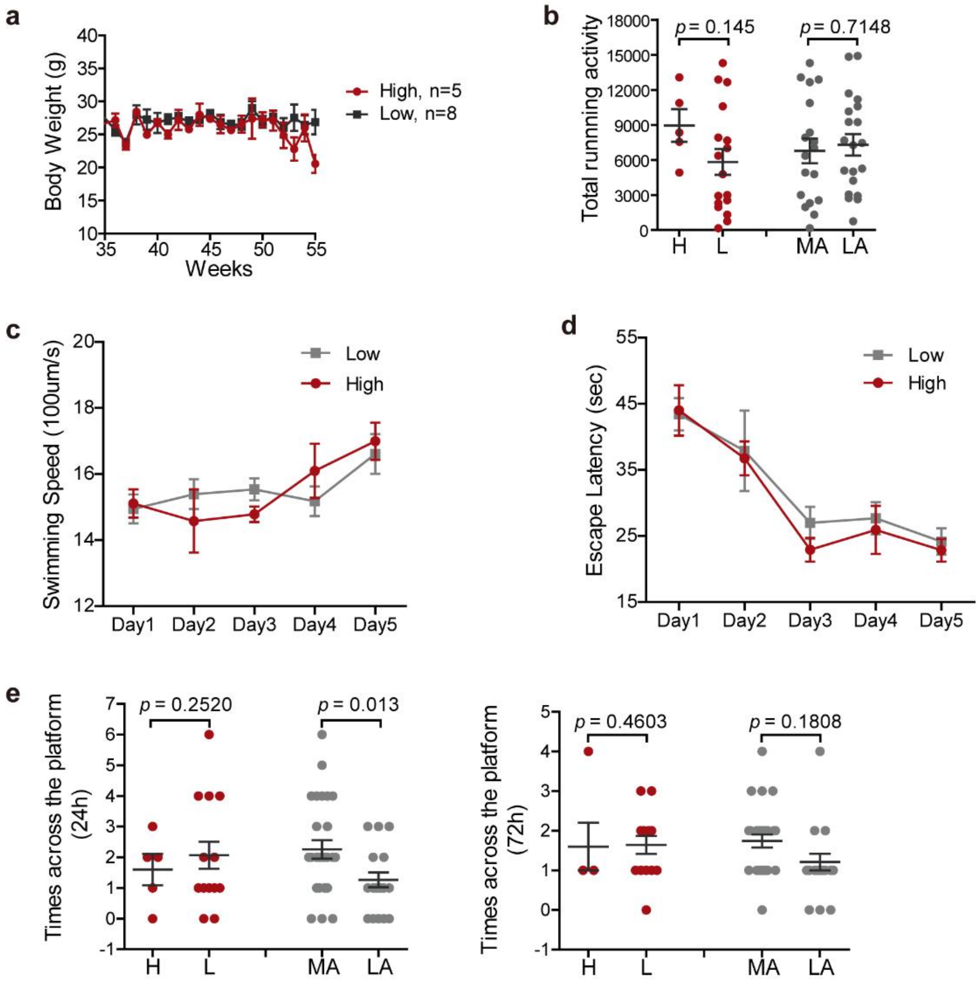

Supplementary Fig. 10 Parameters not correlated with GAC signal

a Body weight of *Glb1*$^{+/m}$ mice with high or low GAC signal (H, n=5, L, n=8). **b** Running activity of *Glb1*$^{+/m}$ mice with high (H) or low (L) GAC signal, and middle-aged (MA) or late-aged (LA) *Glb1*$^{+/m}$ mice (H, n=5, L, n=13, MA, n=18, LA, n=20). **c**, **d** Swimming speed (**c**) and escape latency (**d**) over a 5-day period of Glb1+/m mice with high or low GAC signal in water maze assay (H, n=6, L, n=21). **e** Times across the platform of *Glb1*$^{+/m}$ mice with high (H) or low (L) GAC signal, and middle-aged (MA) or late-aged (LA) *Glb1*$^{+/m}$ mice recorded 24 h (H, n=5, L, n=15, MA, n=27, LA, n=19) and 72 h (H, n=5, L, n=14, MA, n=27, LA, n=19) after training in water maze assay. 'n' represents number of biological replicates. Data represent the means ± s.e.m. *P* value was calculated by Student's *t*-test (two-sided).

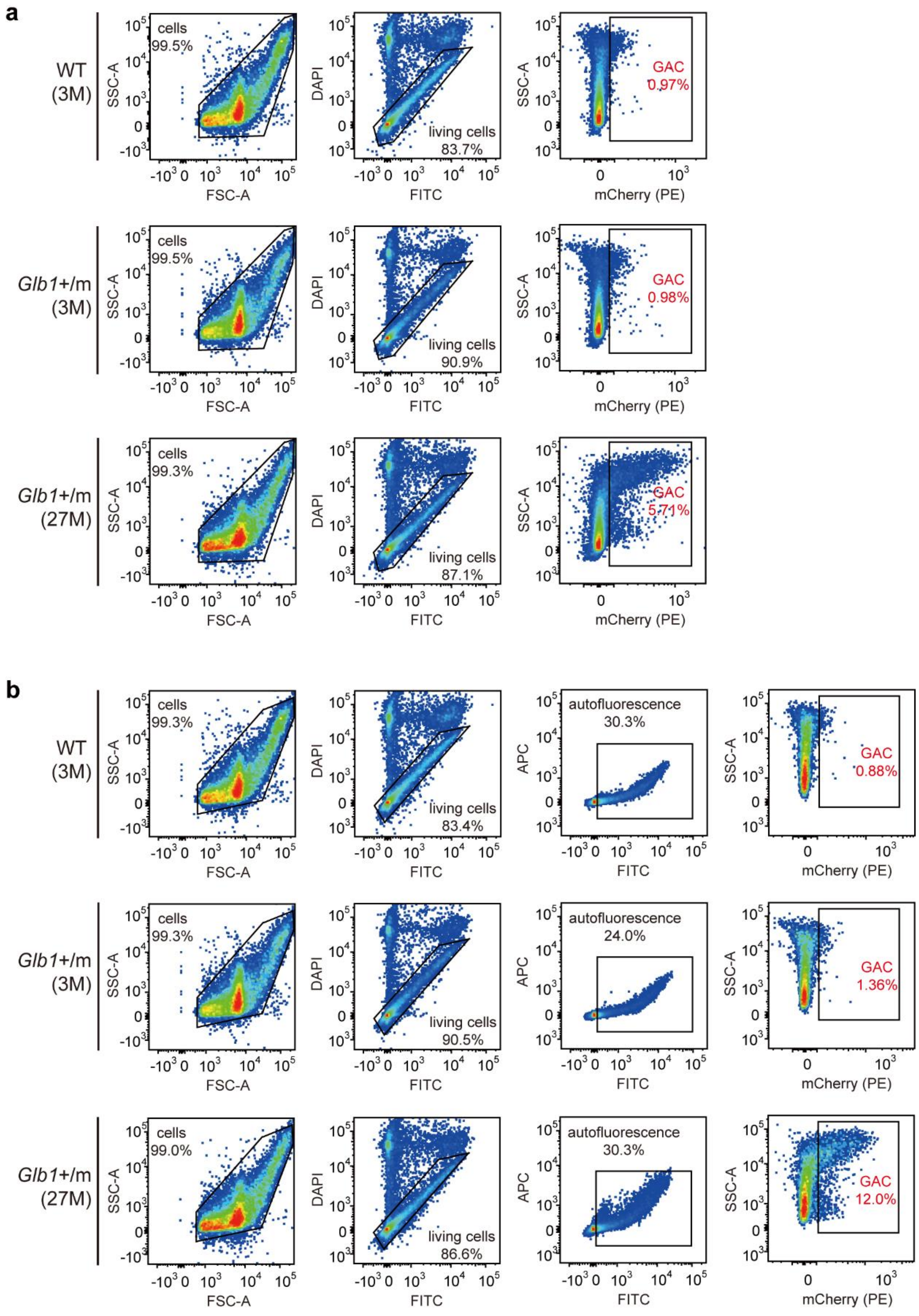

Supplementary Figure 11 GAC signal in cardiomyocytes detected by FACS

a GAC signal in mixed cardiac cells by FACS analysis. **b** GAC signal in gated cardiomyocytes by FACS analysis. WT (wild-type, 3 m, female, n = 3); *Glb1*$^{+/m}$ (3 m, female, n = 3; 27 m, female, n = 3). ‘n’ represents number of biological replicates.

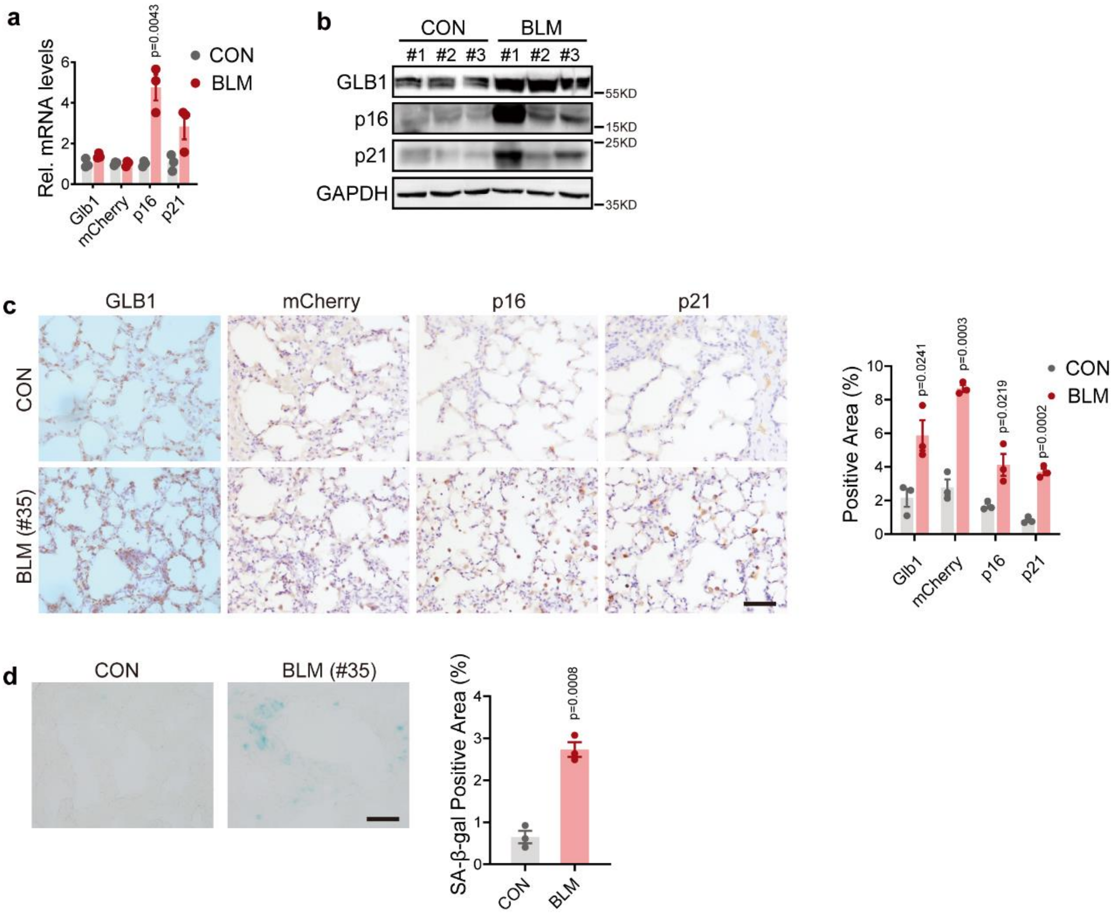

Supplementary Figure 12 Senescence characterization in DOX and BLM-treated *Glb1*$^{+/m}$ mice

a, b The mRNA levels of *Glb1*, *mCherry*, $p16^{Ink4a}$, $p21^{Wif1}$ (**a**) and protein levels of GLB1, p16$^{\text{Ink4a}}$ and p21$^{\text{Wif1}}$ (**b**) in lung sections of BLM-treated (BLM, 16 m, female, n = 3) or untreated (CON, 16 m, female, n = 3) *Glb1*$^{+/m}$ mice. **c** Immunohistochemical staining of GLB1, mCherry, p16$^{\text{Ink4a}}$ and p21$^{\text{Wif1}}$ in lung sections of BLM and CON *Glb1*$^{+/m}$ mice and quantification of staining intensity. Tissues from 3 mice were quantified in each group. **d** SAβ-gal staining of lung tissues of BLM and CON *Glb1*$^{+/m}$ mice and quantification of SAβ-gal positive area. Tissues from 3 mice were quantified in each group. ‘n’ represents number of biological replicates. Scale bar, 50 μm. Data represent the means ± s.e.m. *P* value was calculated by Student’s *t*-test (two-sided).

Supplementary Table 1 Antibodies used in this study

Antibody	Source	Dilutions<br>**WB**, Western blotting;<br>**IF**, immunofluorescence<br>**IHC**, Immunohistochemistry
Lamin B1	Abcam (ab16048)	IF (1:400)
GLB1	GeneTex (GTX134513)	WB (1:1000); IF(1:300)
GLB1	Abcam (ab203749)	IHC (1:150)
p16INK4a	Santa Cruz (sc-1661)	WB (1:200); IHC (1:50); IF(1:50)
p16INK4a	Abcam (ab211542)	WB (1:1000 in MEF)
RFP	Abcam (ab62341)	IF (1:100)
mCherry	Abcam (ab167453)	WB (1:1000); IHC (1:250); IF (1:250)
p21Wif1	Santa Cruz (sc-6246)	WB (1:200); IHC (1:50); IF(1:50 in MEF)
p21Wif1	Abcam (ab188224)	IF (1:300 in tissue)
α-SMA	Sigma-Aldrich (A5228)	IF (1:300)
Ki67	BD (550609)	IF (1:500)
GAPDH	Beyotime (AG019)	WB (1:5000)

Supplementary Table 2 Primers for q-PCR used in this study

**Targets**	**Sequences (5’-3’)**
m*p16*-F	GCCCAACGCCCCGAACTCTTTC
m*p16*-R	GCGACGTTCCCAGCGGTACACA
m*p21*-F	CCTGGTGATGTCCGACCTG
m*p21*-R	CCATGAGCGCATCGCAATC
m*Glb1*-F	CTTCCCACTGAACACTGAGGC
m*Glb1*-R	TTGGCACGAACAAGGTCTTTT
mCherry-F	CCCCGTAATGCAGAAGAAGA
mCherry-R	TTGACCTCAGCGTCGTAGTG
m*Gapdh*-F	CTTTGTCAAGCTCATTTCCTGG
m*Gapdh*-R	TCTTGCTCAGTGTCCTTGC
m*IL6*-F	CTGCAAGAGACTTCCATCCAG
m*IL6*-R	AGTGGTATAGACAGGTCTGTTGG
m*TNFα*-F	CAGGCGGTGCCTATGTCTC
m*TNFα*-R	CGATCACCCCGAAGTTCAGTAG
m*IL1β*-F	GAAATGCCACCTTTTGACAGTG
m*IL1β*-R	TGGATGCTCTCATCAGGACAG
h*Glb1*-F	TATACTGGCTGGCTAGATCACTG
h*Glb1*-R	GGCAAAATTGGTCCCACCTATAA
h*p16*-F	GATCCAGGTGGGTAGAAGGTC
h*p16*-R	CCCCTGCAAACTTCGTCCT
h*p21*-F	TGTCCGTCAGAACCCATGC
h*p21*-R	AAAGTCGAAGTTCCATCGCTC

Uncropped blot images for supplementary figures

Supplementary Figure 2b

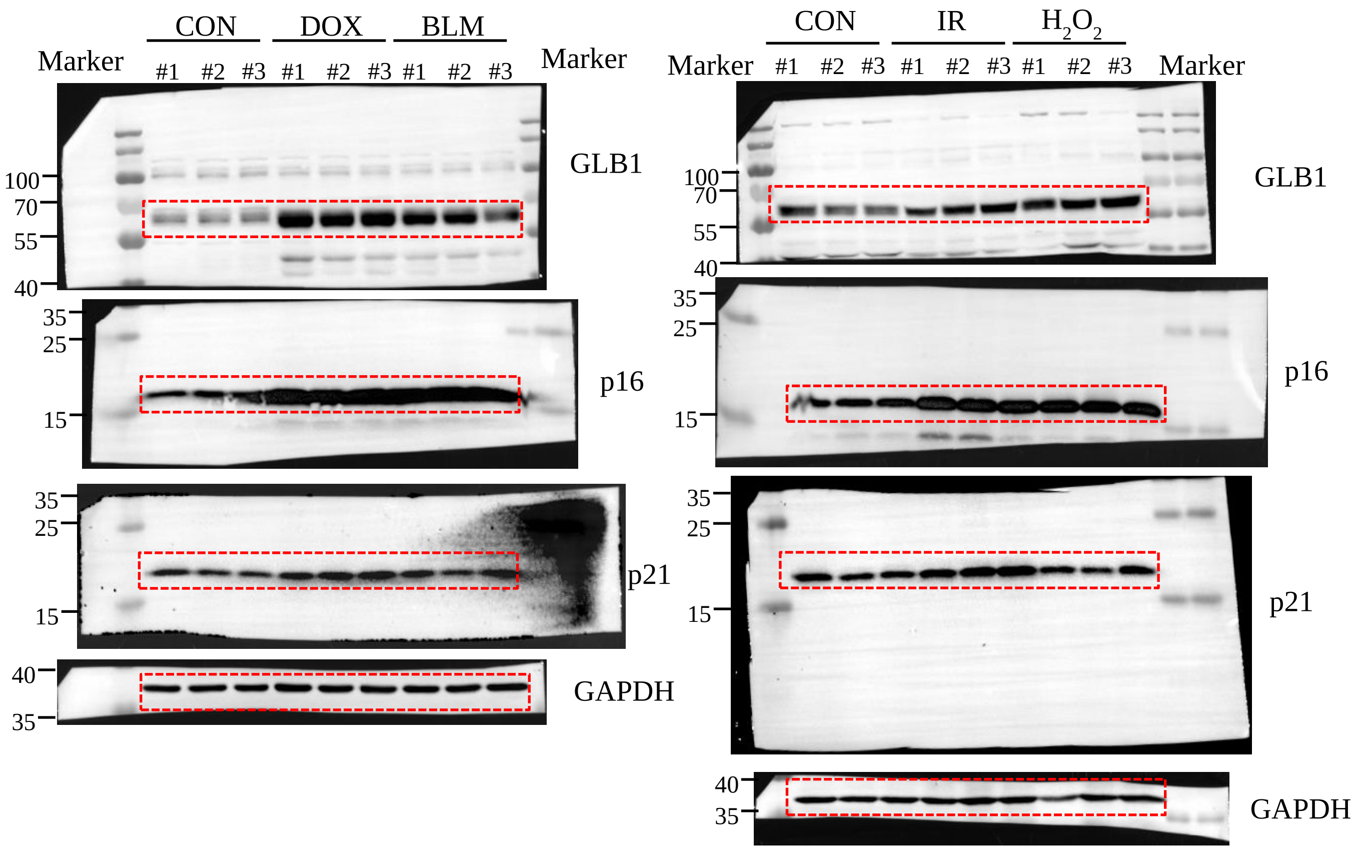

Supplementary Figure 4a

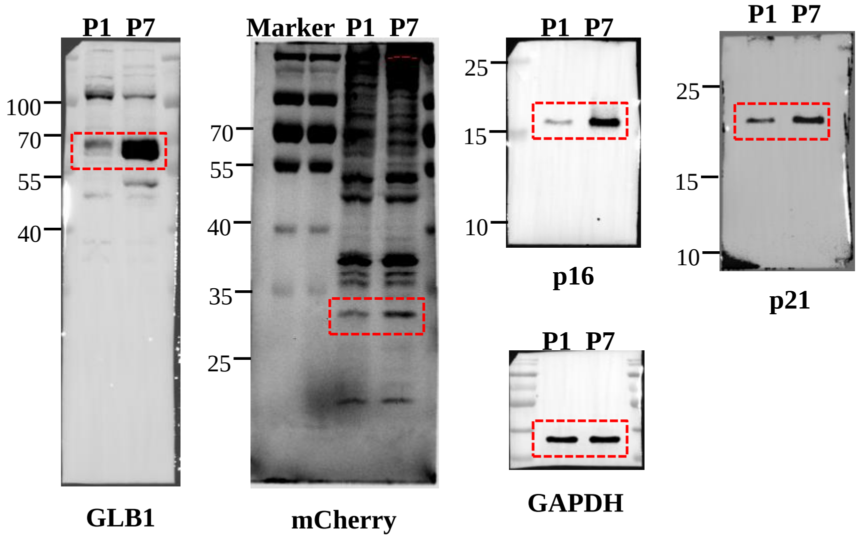

Supplementary Figure 12b

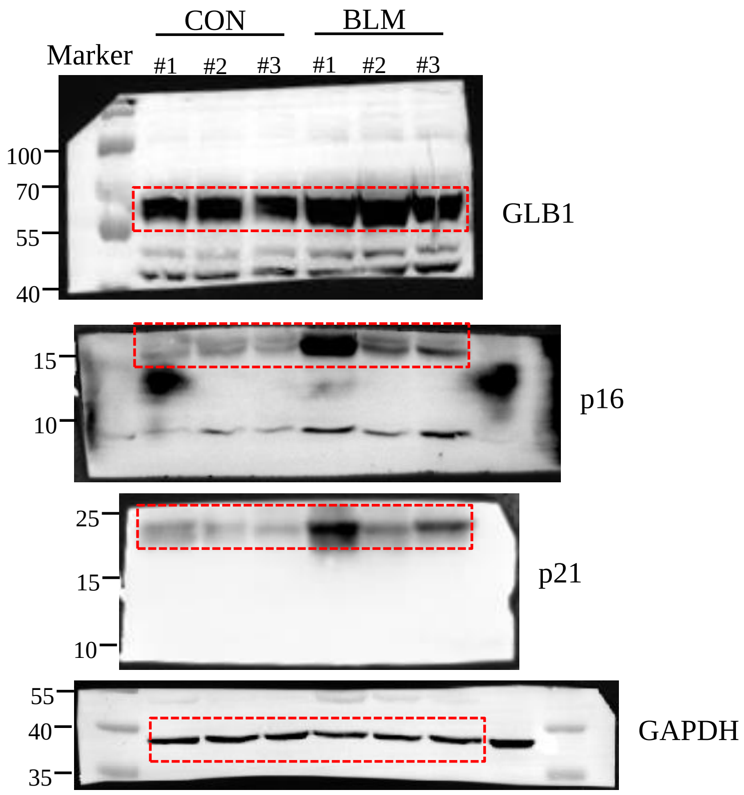

Supplement: Supplementary file 1 — Supplementary information [file 41467_2022_34801_MOESM1_ESM.pdf]
