# Supplementary material for: A Glb1-2A-mCherry reporter monitors systemic aging and predicts lifespan in middle-aged mice
Source: Nat Commun. 2022 Nov 17;13:7028. doi: 10.1038/s41467-022-34801-9 (PMC9671911; doi:10.1038/s41467-022-34801-9)
Supplement: Supplementary file 2 — Reporting Summary [file 41467_2022_34801_MOESM2_ESM.pdf]

nature portfolio

Corresponding author(s): Baohua Liu

Last updated by author(s): Sep 26, 2022

Reporting Summary

Nature Portfolio wishes to improve the reproducibility of the work that we publish. This form provides structure for consistency and transparency in reporting. For further information on Nature Portfolio policies, see our Editorial Policies and the Editorial Policy Checklist.

Statistics

For all statistical analyses, confirm that the following items are present in the figure legend, table legend, main text, or Methods section.

n/a	Confirmed	
☐	☒	The exact sample size (*n*) for each experimental group/condition, given as a discrete number and unit of measurement
☐	☒	A statement on whether measurements were taken from distinct samples or whether the same sample was measured repeatedly
☐	☒	The statistical test(s) used AND whether they are one- or two-sided<br>*Only common tests should be described solely by name; describe more complex techniques in the Methods section.*
☒	☐	A description of all covariates tested
☒	☐	A description of any assumptions or corrections, such as tests of normality and adjustment for multiple comparisons
☐	☒	A full description of the statistical parameters including central tendency (e.g. means) or other basic estimates (e.g. regression coefficient) AND variation (e.g. standard deviation) or associated estimates of uncertainty (e.g. confidence intervals)
☐	☒	For null hypothesis testing, the test statistic (e.g. *F*, *t*, *r*) with confidence intervals, effect sizes, degrees of freedom and *P* value noted<br>*Give P values as exact values whenever suitable.*
☒	☐	For Bayesian analysis, information on the choice of priors and Markov chain Monte Carlo settings
☒	☐	For hierarchical and complex designs, identification of the appropriate level for tests and full reporting of outcomes
☒	☐	Estimates of effect sizes (e.g. Cohen's *d*, Pearson's *r*), indicating how they were calculated

Our web collection on statistics for biologists contains articles on many of the points above.

Software and code

Policy information about availability of computer code

Data collection	Immunoblots data were collected by ChemiDoc Touch Imaging System (BIO-RAD).<br>IHC and SAβ-gal staining images were collected by BX43 (OLYMPUS)<br>Real-time PCR data were collected by BIO-RAD CFX Connect.<br>Flow cytometry data were collected by FACS Aria II (BD bioscience, USA).<br>Immunofluorescent images were collected by DragonFly confocal imaging system (Andor Technology)<br>The echocardiographic evaluation data were collected by Vevo 2100 Imaging System (Visual Sonics).<br>The data of morris water maze assay were collected by WaterMaze3 Tracking system (Actimetrics).<br>The in vivo living imaging data were collected by IVIS Lumina II system (Caliper Life Sciences)
Data analysis	Image Lab Software V5.2.1 build 11 (BIO-RAD) was used for immunoblotting analyses.<br>Excel (Microsoft 365 Family) and GraphPad Prism 9.2.0 software were used for statistical analyses.<br>Image J software was used for quantification analyses (V1.52).<br>FlowJo_V10 software was used for flow cytometry data analyses.<br>Imaris Viewer x64 9.7.0 software was used for immunofluorescent images analyses.<br>Vevo 2100 Workstation software was used for echocardiographic evaluation data analyses.<br>WaterMaze software was used for water maze assay data analyses.<br>Living Image Software (Caliper Life Sciences) was used for IVIS data analyses.

For manuscripts utilizing custom algorithms or software that are central to the research but not yet described in published literature, software must be made available to editors and reviewers. We strongly encourage code deposition in a community repository (e.g. GitHub). See the Nature Portfolio guidelines for submitting code & software for further information.

Data

Policy information about availability of data

All manuscripts must include a data availability statement. This statement should provide the following information, where applicable:

- Accession codes, unique identifiers, or web links for publicly available datasets
- A description of any restrictions on data availability
- For clinical datasets or third party data, please ensure that the statement adheres to our policy

Other data that supports the findings of this study are available in the supplementary information of this article. Source data are provided with this paper.

Field-specific reporting

Please select the one below that is the best fit for your research. If you are not sure, read the appropriate sections before making your selection.

☒ Life sciences ☐ Behavioural & social sciences ☐ Ecological, evolutionary & environmental sciences

For a reference copy of the document with all sections, see nature.com/documents/nr-reporting-summary-flat.pdf

Life sciences study design

All studies must disclose on these points even when the disclosure is negative.

Sample size

The sample size was not statistically predetermined. The sample for q-PCR, western blots, IHC, IF analyses were harvested from at least three biological replicates and the sample size were chosen in agreement with common practice in the field (Wei Wang et al., 2021; Yi Bao et al., 2020; Stephanie P. et al., 2021). For the IVIS, echocardiographic evaluation, lifespan and water maze analysis,the sample size was determined on the sample availability (more is better) and the samples sizes were similar to those generally employed and accepted in the field (Burd, C.E. et al., 2013; Zhonghao Zhang et al., 2017; Shimin Sun et al., 2020). All sample sizes are shown in the figure legends or under the methods section of our manuscript, respectively.

Data exclusions

The mice unsuitable for IVIS imaging owing to large area of black coat after shaving are excluded for analysis. The mice with cataract or obvious eye disease were excluded in the water maze assay.

Replication

All experiments were performed with at least three biological replicates and confirmed from more than two independent experiments.

Randomization

The age-, gender- and genotype-matched mice and cell samples were randomly assigned to experimental groups. For microscopy imaging, and the images were collected randomly from at least 5 fields for each sample. For the senolytic DQ treatment assay, the BLM-treated mice were randomize into different experimental groups.

Blinding

The echocardiographic evaluation assay was blind. For other assay, the data collection and analysis were not performed blind due to obvious differences between groups (e.g. different ages (sizes) of mice, or different genotypes). However, two or more observers performed the experiments and independently analyzed the data.

Reporting for specific materials, systems and methods

We require information from authors about some types of materials, experimental systems and methods used in many studies. Here, indicate whether each material, system or method listed is relevant to your study. If you are not sure if a list item applies to your research, read the appropriate section before selecting a response.

Materials & experimental systems

n/a	Involved in the study
☐	☒ Antibodies
☒	☐ Eukaryotic cell lines
☒	☐ Palaeontology and archaeology
☐	☒ Animals and other organisms
☒	☐ Human research participants
☒	☐ Clinical data
☒	☐ Dual use research of concern

Methods

n/a	Involved in the study
☒	☐ ChIP-seq
☐	☒ Flow cytometry
☒	☐ MRI-based neuroimaging

Antibodies

Antibodies used

Lamin B1, Abcam (ab16048), IF (1:400);
GLB1, GeneTex (GTX134513), WB/ IF (1:1000/1:300);
GLB1, Abcam (ab203749), IHC (1:150);

p16INK4a, Santa Cruz Biotechnology (sc-1661), WB/IHC/ IF (1:200/1:50/1:50);
p16INK4a, abcam (ab211542), WB (1:1000 in MEF);
RFP, abcam (ab62341), IF (1:100);
mCherry, abcam (ab167453), WB/IHC/IF (1:1000/1:250/1:250);
p21Wif1, Santa Cruz Biotechnology(sc-6246), WB/IHC/IF (1:200/1:50/1:50 in MEF);
p21Wif1, abcam(ab188224), IF (1:300 in tissue);
α-SMA, Sigma-Aldrich (A5228), IF (1:300);
Ki67, BD Biosciences (550609), IF(1:500)
GAPDH, Beyotime (AG019), WB (1:5000).

Validation

All these antibodies below are commercially available and all validated by the producers.
Lamin B1, Abcam (ab16048), IF (1:400);
https://www.abcam.cn/lamin-b1-antibody-nuclear-envelope-marker-ab16048.html;
GLB1, GeneTex (GTX134513), WB/ IF (1:1000/1:300);
http://www.neobioscience.com/prod_view.aspx?TypeId=223&Id=891163&FId=t3:223:3;
GLB1, Abcam (ab203749), IHC (1:150);
https://www.abcam.cn/glb1beta-galactosidase-antibody-ab203749.html;
p16INK4a, Santa Cruz Biotechnology (sc-1661), WB/IHC/ IF (1:200/1:50/1:50);
https://www.scbt.com/p/p16-antibody-f-12?requestFrom=search;
p16INK4a, abcam (ab211542), WB (1:1000 in MEF);
https://www.abcam.cn/cdkn2ap16ink4a-antibody-epr20418-ab211542.html;
RFP, abcam (ab62341), IF (1:100);
https://www.abcam.cn/rfp-antibody-ab62341.html;
mCherry, abcam (ab167453), WB/IHC/IF (1:1000/1:250/1:250);
https://www.abcam.cn/mcherry-antibody-ab167453.html;
p21Wif1, Santa Cruz Biotechnology(sc-6246), WB/IHC/IF (1:200/1:50/1:50 in MEF);
https://www.scbt.com/p/p21-antibody-f-5?requestFrom=search;
p21Wif1, abcam(ab188224), IF (1:300 in tissue);
https://www.abcam.cn/p21-antibody-epr18021-ab188224.html;
α-SMA, Sigma-Aldrich (A5228), IF (1:300);
https://www.sigmaaldrich.cn/CN/zh/product/sigma/a5228;
Ki67, BD Biosciences (550609), IF(1:500);
https://www.bdbiosciences.com/en-us/products/reagents/flow-cytometry-reagents/research-reagents/single-color-antibodies-ruo/purified-mouse-anti-ki-67.550609;
GAPDH, Beyotime (AG019), WB (1:5000).
https://www.beyotime.com/product/AG019.htm.

Animals and other organisms

Policy information about studies involving animals; ARRIVE guidelines recommended for reporting animal research

Laboratory animals

Glb1+/m mice, C57BL6/J background, 1~27 months, males and females.
The Glb1+/m mouse model (Glb1-2A-mCherry knock-in allele) in C57BL6/J background was generated using the CRISPR/Cas9 system by Shanghai Biomodel Organism Science & Technology Development Co. Ltd. (Shanghai, China). Briefly, the coding sequences of 2A peptide and mCherry were sequentially added to the 3′ end of the Glb1 open reading frame (ORF), thus generating a new ORF encoding a Glb1-2A-mCherry fused peptide.
The Glb1+/m mice were intercrossed to produce the Glb1+/+ and Glb1m/m mice, and the wild-type Glb1+/+ littermates were used as the control. Mice were maintained at 21-23 °C , in 40% to 60% humidity, and with a 12 h light/12 h dark light cycle.

Wild animals

No wild animals were used in this study.

Field-collected samples

This study did not involve any field-collected samples.

Ethics oversight

Animal experiments were conducted in accordance with ethical and scientific protocols approved by the Committee on the Use of Live Animals in Teaching and Research of Shenzhen University, China.

Note that full information on the approval of the study protocol must also be provided in the manuscript.

Flow Cytometry

Plots

Confirm that:

☒ The axis labels state the marker and fluorochrome used (e.g. CD4-FITC).

☒ The axis scales are clearly visible. Include numbers along axes only for bottom left plot of group (a 'group' is an analysis of identical markers).

☒ All plots are contour plots with outliers or pseudocolor plots.

☒ A numerical value for number of cells or percentage (with statistics) is provided.

Methodology

Sample preparation

For cells: MEF cells were incubated with 20 μM of C12FDG for 0.5~1 h and HuFB cells were incubated with 33 μM of C12FDG

Sample preparation	for 1.5~2 h. Cells were washed with PBS and harvested for flow cytometry analysis.<br>For tissues: After mice were euthanized, the hearts were perfused with pre-cold perfusion buffer and then anterior wall of left ventricle was removed to digestion buffer in 37 °C shaking water bath for 15 min. After digestion of cardiac tissue, the cell lysate passed through a 100 µm cell strainer and cells we collected were resuspended in DMEM with DAPI for flow cytometry analysis.
Instrument	The flow cytometry was performed by FACS Aria II (BD bioscience, USA).
Software	FlowJo_V10 software was used for flow cytometry data analyses.
Cell population abundance	83~90% of cardiac cells were identified as live and single cell. ~30% cardiac cells were identified as cardiomyocytes (consistent with the study of Monika M. Gladka et al., 2018), and ~6% mCherry-positive cardiac cells while ~12% mCherry-positive cardiomyocytes were identified.
Gating strategy	Forward scatter area (FSC-A) and side scatter area (SSC-A) was used to sort for cells, DAPI negative was used to sort for living cells, and PE channel was used to sort for mCherry-positive cardiac cells. For sorting mCherry-positive cardiomyocytes, we employed additional FITC channel to sort for cardiomyocytes (with green autofluorescence) before PE channel selection.<br>For sorting C12FDG-positive MEF or human fibroblasts, the FITC channel was used to gate based on the correspondingly unstained cell sample.

☒ Tick this box to confirm that a figure exemplifying the gating strategy is provided in the Supplementary Information.
