# Supplementary figures and images for: A Glb1-2A-mCherry reporter monitors systemic aging and predicts lifespan in middle-aged mice
Source: Nat Commun. 2022 Nov 17;13:7028. doi: 10.1038/s41467-022-34801-9 (PMC9671911; doi:10.1038/s41467-022-34801-9)

### Figure 1c

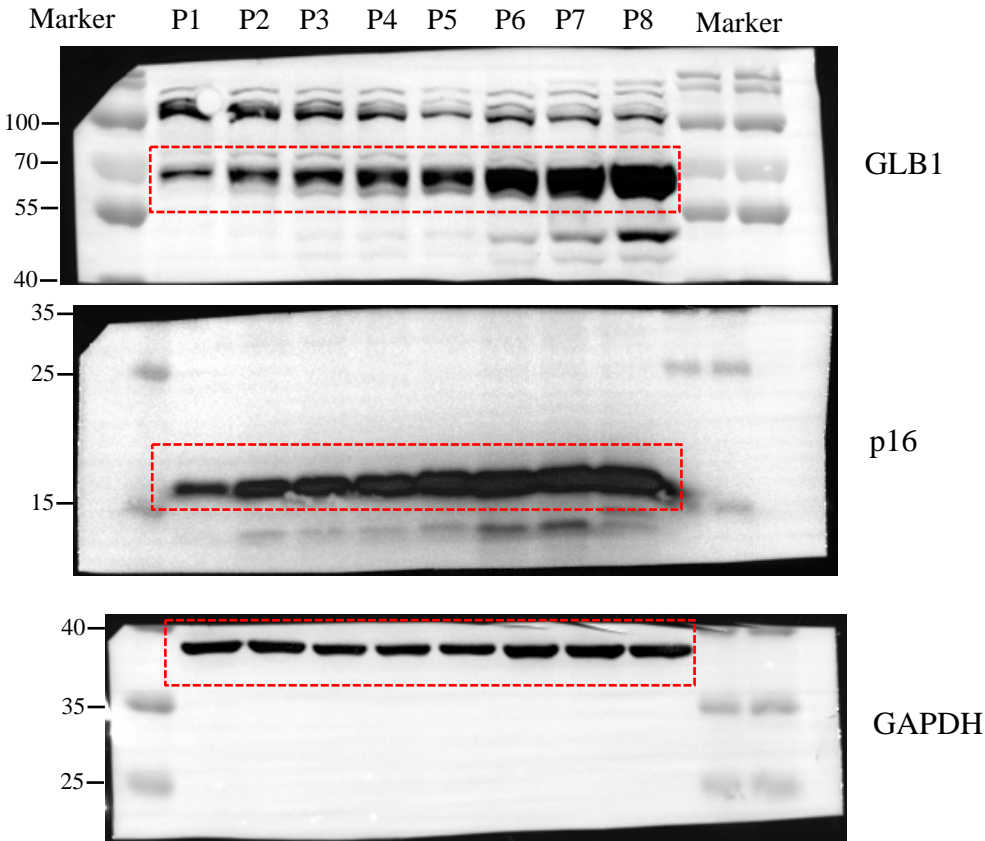

Figure 1g

Brain

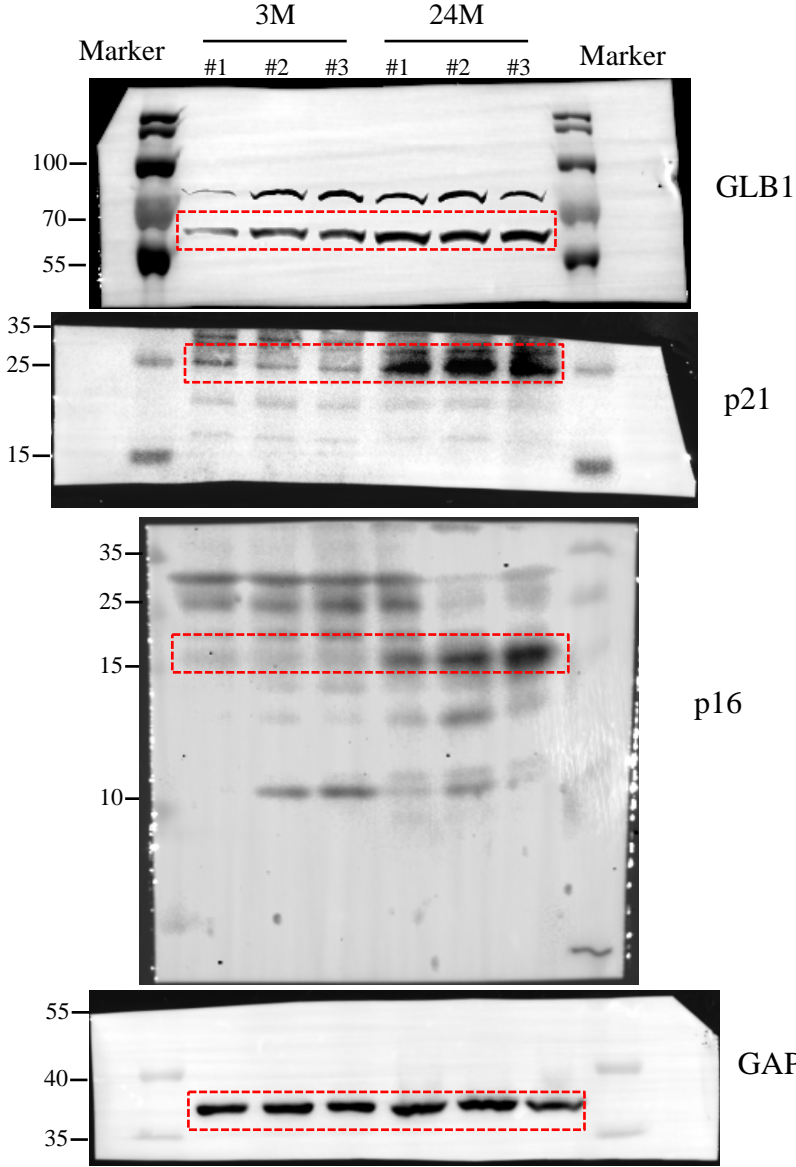

Heart

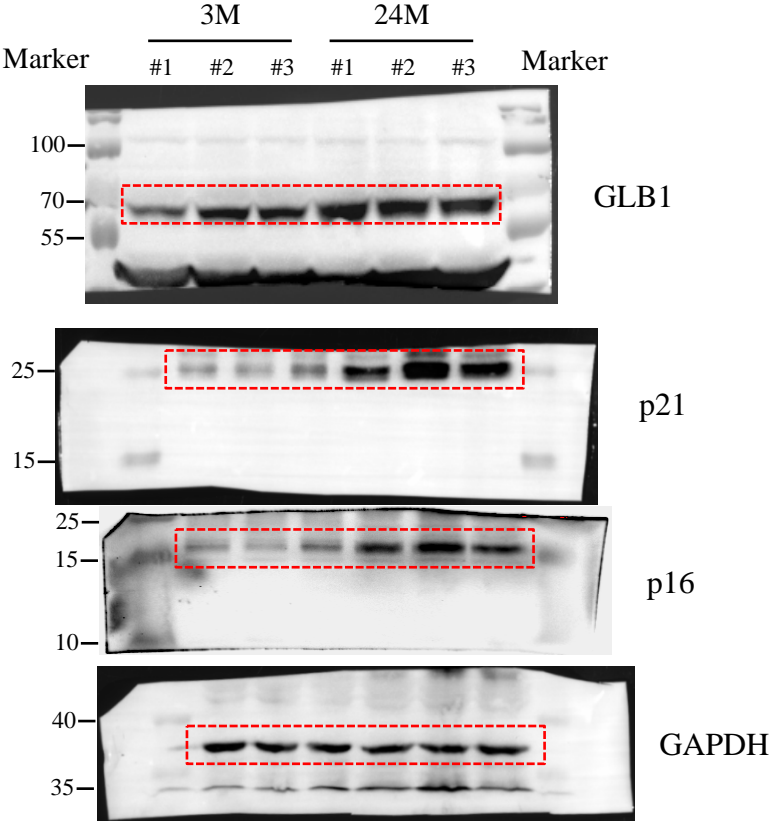

**Figure 1g**

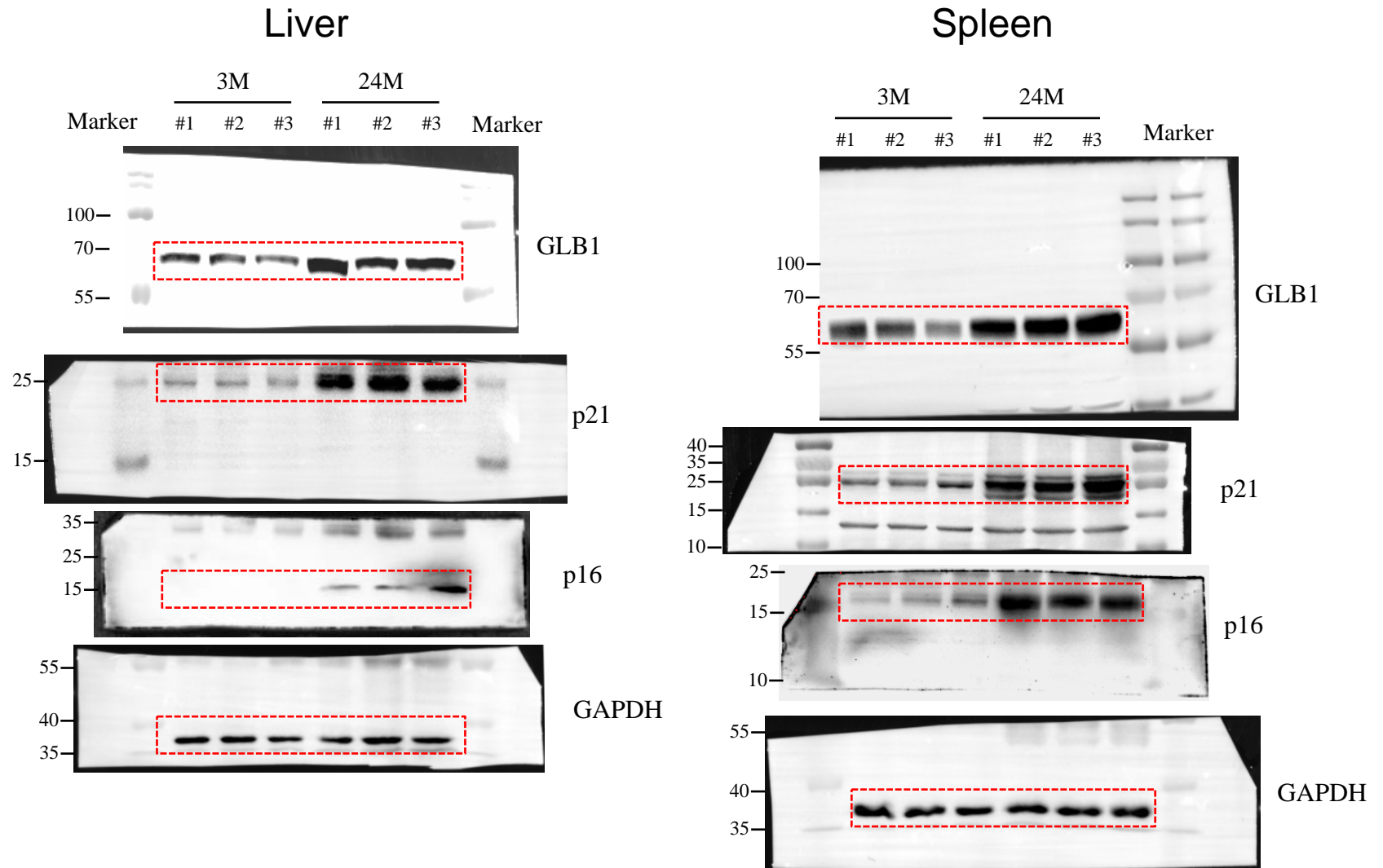

**Figure 1g**

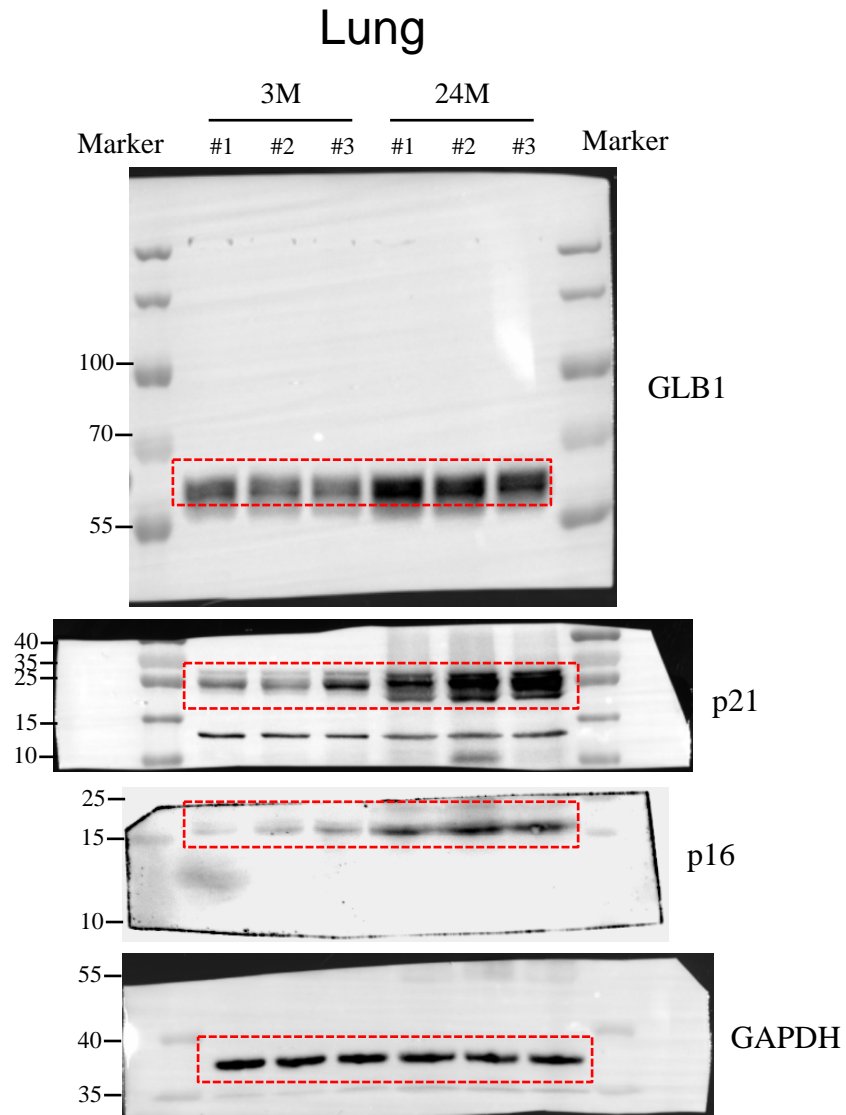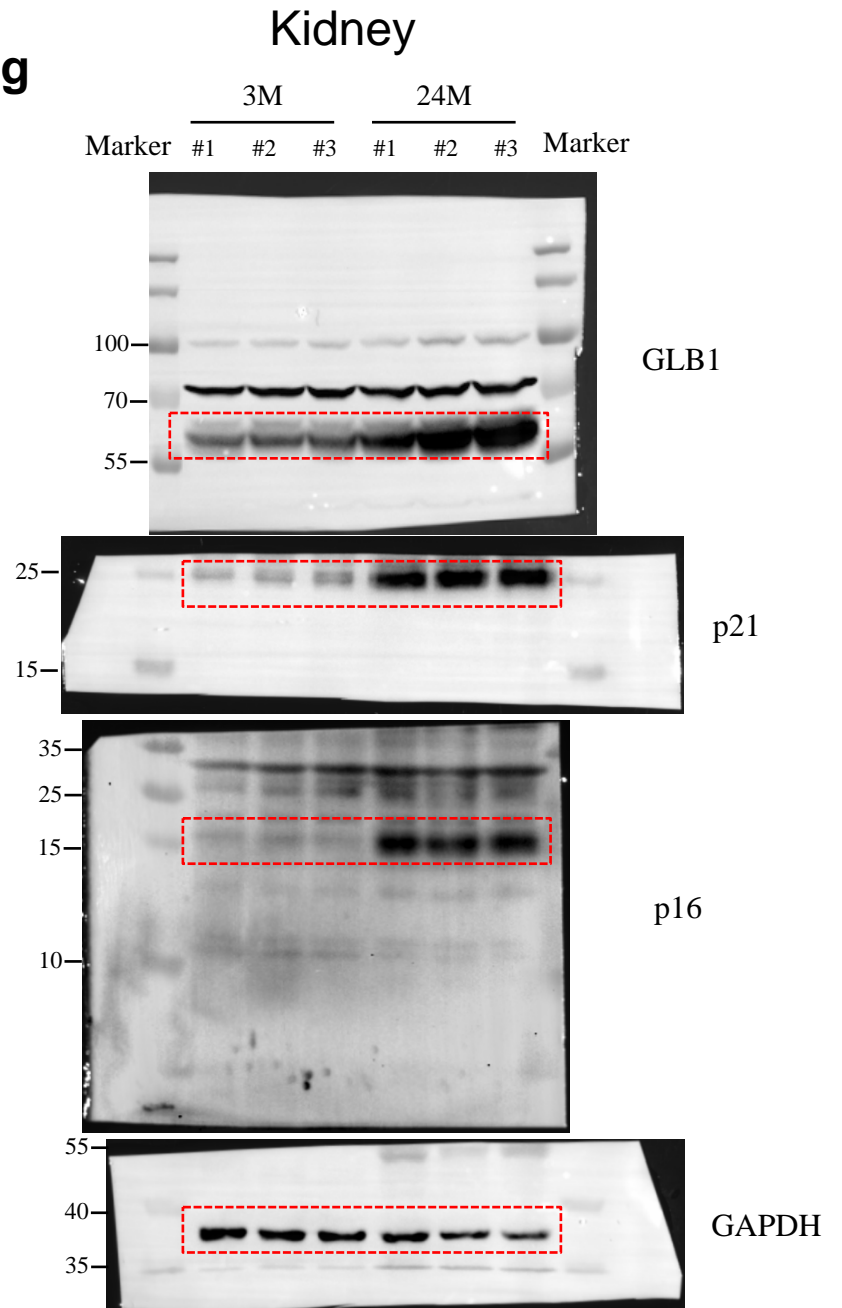

**Figure 2b**

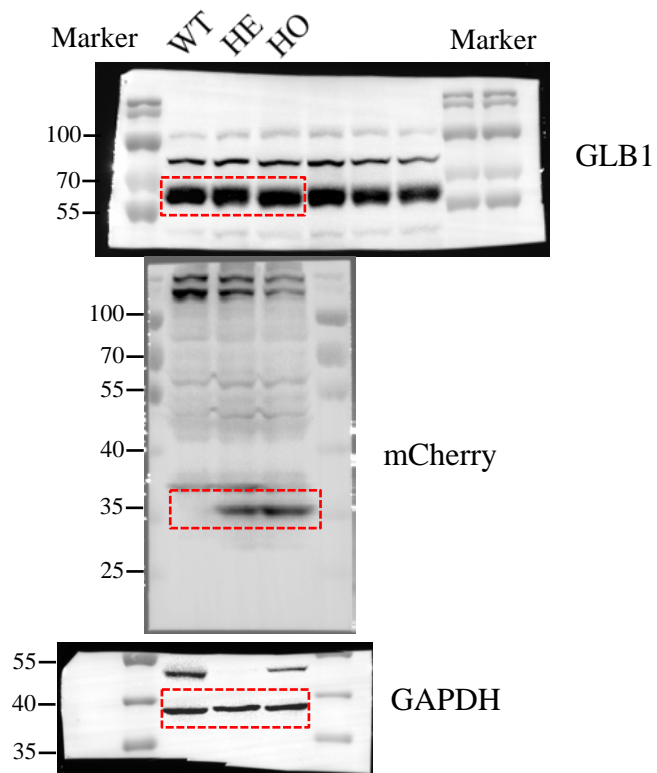

**Figure 2h**

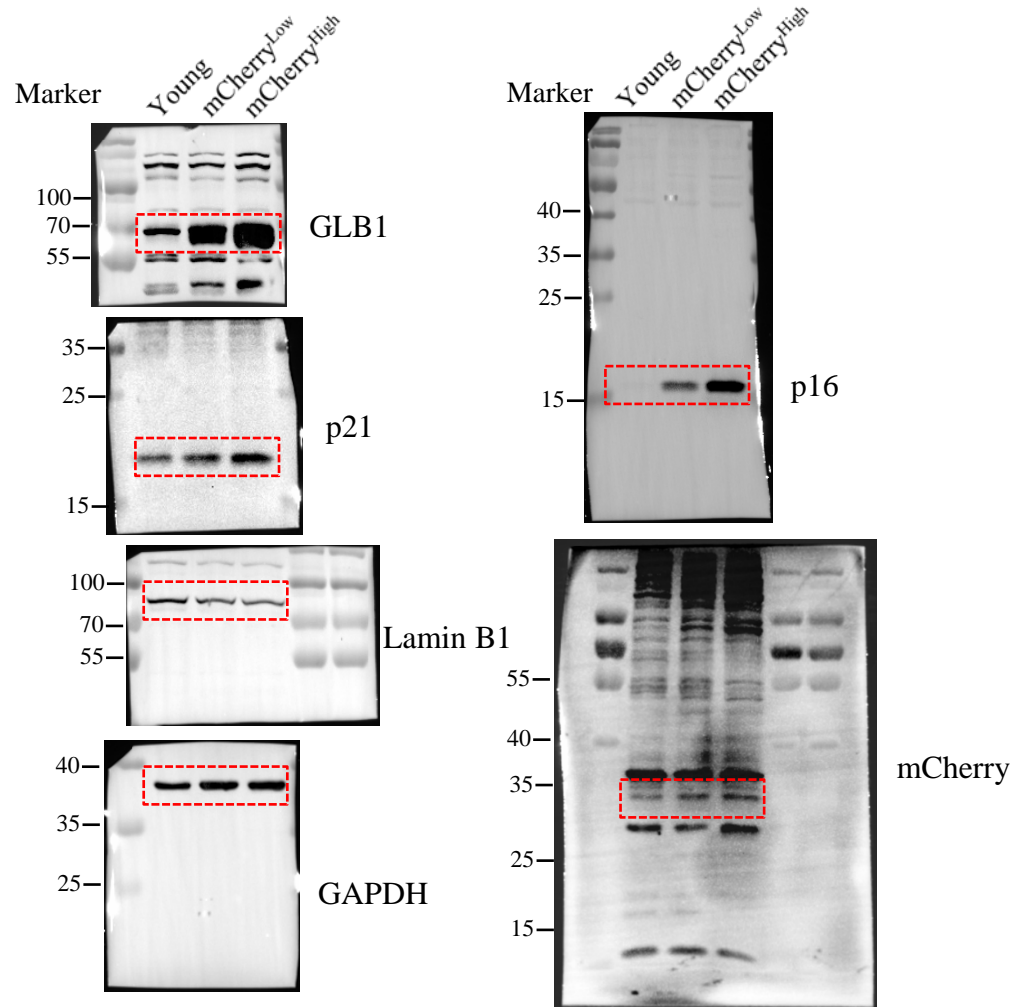

# Figure 7e

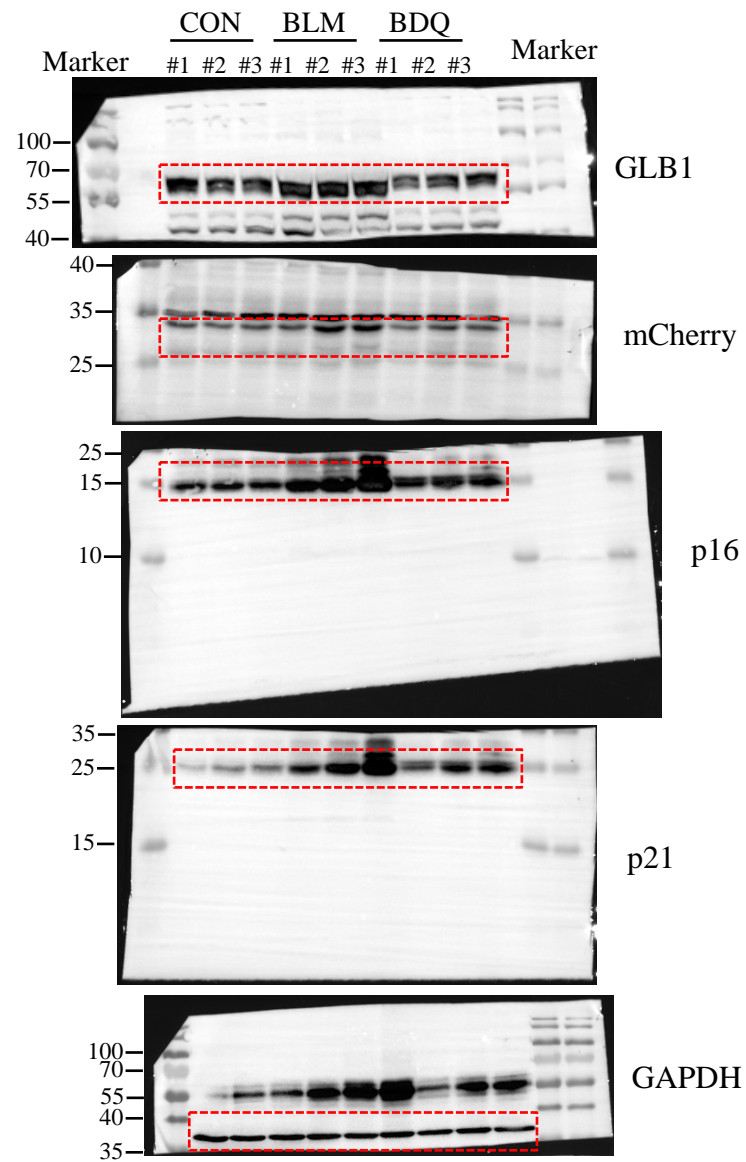

Supplement: Supplementary file 3 — Source Data [file 41467_2022_34801_MOESM3_ESM.zip › Source data-Uncropped scans.pdf]
